# Long‐term effects of non‐pharmacological interventions in adolescents and young adults with type 1 diabetes: A systematic review and meta‐analysis

**DOI:** 10.1111/wvn.12751

**Published:** 2024-11-04

**Authors:** DaeEun Lee, Haejung Lee, Misoon Lee, Gaeun Park

**Affiliations:** ^1^ College of Nursing Pusan National University Yangsan‐si South Korea; ^2^ College of Nursing/Research Institute of Nursing Science Pusan National University Yangsan‐si South Korea; ^3^ Department of Nursing Youngsan University Yangsan Campus Yangsan‐si South Korea

**Keywords:** adolescent, diabetes mellitus type 1, follow‐up studies, meta‐analysis, randomized controlled trial, young adult

## Abstract

**Background:**

Consistent diabetes control is crucial for patients with type 1 diabetes (T1D) to prevent diabetic complications. Analyzing the long‐term effects of non‐pharmacological interventions can improve diabetes management.

**Aim:**

To examine the long‐term effects of non‐pharmacological interventions in adolescents and young adults with T1D through a systematic review and meta‐analysis of randomized controlled trials (RCTs).

**Methods:**

The searches, without any year limitations, were conducted in seven databases. Two reviewers independently performed data extraction and risk of bias assessments. The meta‐analysis was conducted using the RevMan 5.4 program and R Studio. The study was registered with PROSPERO (CRD42024503185).

**Results:**

A total of 40 studies were included in the systematic review, of which 28 were included in the meta‐analysis. Notably, non‐pharmacological interventions were effective in improving glycated hemoglobin (HbA1c) at 6 months and self‐care at >12 months of follow‐up. Subgroup analyses revealed that combined interventions encompassing self‐care behavior management, education, and psychological interventions could enhance self‐care. Additionally, a combination of face‐to‐face and telephonic communication was effective in improving HbA1c.

**Linking Evidence to Action:**

Non‐pharmacological interventions are effective in improving HbA1c levels and self‐care in adolescents and young adults undergoing long‐term treatment. However, few studies have analyzed their effects on cardiovascular disease risk factors. Future studies should investigate the long‐term effects of non‐pharmacological interventions.

## INTRODUCTION

According to the 2022 International Diabetes Federation statistics, the number of people with type 1 diabetes (T1D) has reached 8.75 million worldwide, of which 1.52 million (17.4%) are aged <20 years (Ogle et al., 2022). Managing blood glucose levels in patients with T1D is critical. However, achieving glycemic control is challenging for adolescents and young adults with T1D (Holman et al., 2023). A cohort study with a 10‐year follow‐up involving patients with T1D revealed a nine‐fold higher incidence of coronary artery disease in patients with T1D compared to the general population (Rawshani et al., 2018). Adolescents with T1D are exposed to risky behaviors, such as tobacco and alcohol use (Yetim et al., 2018), and face additional self‐care challenges specific to T1D, including gaining independence from their parents, adapting to adult roles, completing high school and university, entering the workforce, and managing romantic relationships (Carlsund & Söderberg, 2018; Saylor et al., 2019). The highest levels of glycated hemoglobin (HbA1c) and hospitalization rates for diabetic ketoacidosis are observed in individuals aged 17–19, with both decreasing after the age of 30 (Holman et al., 2023). Strategies to prevent and mitigate diabetic complications in adolescents with T1D are necessary and assessing the long‐term effects of intervention studies is essential for developing effective long‐term strategies.

Between 2011 and 2024, nine meta‐analyses have been published reviewing the effectiveness of non‐pharmacological interventions in individuals with T1D. These interventions, including physical activity, psychological interventions, telecare, educational interventions, mobile app interventions, diet, and behavioral therapies, have been shown to effectively improve HbA1c levels, muscle strength, lipid profiles, quality of life, and anxiety (Charalampopoulos et al., 2017; de Abreu de Lima et al., 2022; Hou et al., 2016; Liu et al., 2020; Muñoz‐Pardeza et al., 2024; Quirk et al., 2014; Viana et al., 2016; Wu et al., 2019). However, comparing the effectiveness of these interventions for improving diabetes management and preventing complications in adolescents and young adults with T1D remains challenging due to the limited number of studies examining their long‐term effects, insufficient subgroup analyses, and a narrow range of intervention types and outcome variables.

For instance, the effects of non‐pharmacological interventions were reviewed in the meta‐analyses conducted by Lee et al. (2024) and Muñoz‐Pardeza et al. (2024); however, these studies primarily focused on pre‐ and post‐intervention effects related to diabetes complications. Subgroup analyses conducted by Lee et al. (2024) examined the type and duration of interventions to identify those most effective in improving HbA1c levels. In contrast, Muñoz‐Pardeza et al. (2024) focused on identifying the most effective interventions for improving both HbA1c levels and daily insulin dosage immediately following the intervention. Neither study assessed the long‐term effects of these interventions. The short term effects of structured educational intervention (Liu et al., 2020), psychoeducational interventions (Charalampopoulos et al., 2017; Viana et al., 2016), mobile app interventions (Wu et al., 2019), physical activity intervention (Quirk et al., 2014), and life style modification (Wu et al., 2019) were reviewed. Wu et al. (2019) identified only one study that assessed long‐term effectiveness. Additionally, subgroup analyses were conducted on the type of intervention (Charalampopoulos et al., 2017; Viana et al., 2016) and on age group and follow‐up duration (Liu et al., 2020).

These limitations necessitate a comprehensive review of the long‐term effects of non‐pharmacological interventions. Additionally, subgroup analyses based on the type, frequency, and duration of the interventions could offer more detailed insights into their effectiveness and enhance their applicability. Consequently, updated evaluations are essential to determine whether different non‐pharmacological interventions lead to better outcomes in adolescents and young adults with T1D. The purpose of this study is to systematically review and meta‐analyze previously published research to identify the long‐term effectiveness of non‐pharmacological interventions in preventing diabetes complications in adolescents and young adults with T1D. Our findings aim to inform the development of intervention programs designed to improve diabetes management in this population.

## METHODS

### Research design

This study was conducted following the Preferred Reporting Items for Systematic Reviews and Meta‐analyses guidelines (Higgins et al., 2022) and the Preferred Reporting Items for Systematic reviews and Meta‐Analysis statement (Page et al., 2021) (Appendix S1). The review protocol was registered in PROSPERO (CRD42024503185). The primary research question guiding this systematic review and meta‐analysis was: “What are the long‐term effects of non‐pharmacological interventions on the prevention and management of diabetes complications in adolescents and young adults with T1D?” As this meta‐analysis synthesized data from previously published studies and did not involve any new data collection or direct interaction with the study participants, it was exempted from review by the institutional review board of the researchers' university.

### Eligibility criteria

All peer‐reviewed articles were included, and eligibility criteria were specified based on the population, intervention, comparator, outcome, and study design (PICOS) framework for the following study characteristics: (1) P: adolescents and young adults with T1D, with a mean age or inclusion criteria age of 13–24 in accordance with definition in Medical Subject Headings (MeSH); (2) I: non‐pharmacological interventions for diabetes management, including self‐care behavioral management (e.g., blood glucose monitoring), educational interventions, and psychological interventions (e.g., stress management); (3) C: standard/routine care, usual care, no intervention, or alternative group; (4) O: all psychological and physiological variables quantitatively measured and relevant to the management of diabetes and its associated complications; (5) S: randomized controlled trials (RCTs).

The exclusion criteria were as follows: (1) not published in English or Korean, (2) duplicate studies, (3) conference/presentation abstracts or unpublished literature, (4) interventions not related to diabetes management or non‐pharmacological interventions, such as preconception programs or medication, (5) a follow‐up period of <6 months, and (6) studies in which the interventions' outcomes were not measured.

### Search strategy

A systematic search for eligible articles was conducted in PubMed, Cochrane, CINAHL, Embase, and three Korean databases (RISS, NDSL, and KoreaMed) between December 2023 and February 2024. Search terms included “type 1 diabetes” (type 1 diabetes* OR insulin‐dependent diabetes*), “adolescents” (adolescent* OR teen* OR young*), and “RCTs,” based on the PICOS framework. Relevant literature was retrieved from each database after confirming synonyms and related terms. The MeSH terms in PubMed, Subject Headings in CINAHL, and Emtree in Embase were utilized. Non‐pharmacological interventions, long‐term effects of 6 months or more, and outcome variables related to diabetic complications were manually selected by researchers 1 and 2 through a review of literature titles, abstracts, and full texts. The approach to literature identification is detailed in Appendix S2.

### Study selection

The duplicate articles retrieved from the databases were removed using the EndNote program (Clarivate Analytics, Philadelphia, Pennsylvania, USA). Researcher 1 reviewed the titles and abstracts for the inclusion assessment. Thereafter, researchers 1 and 2 independently reviewed the full‐text articles. Any disagreements between the two researchers were resolved by researcher 3.

### Data extraction

Data were extracted independently by researchers 1 and 2 using Excel 2016. For the systematic review, data were extracted from the studies including the name of the first author, publication year, country, sample size, mean age of the participants, age inclusion criteria, duration of T1D diagnosis, ethnicity/race, types, frequency, and duration of interventions, outcome variables and their values, and follow‐up duration. For the meta‐analysis, medians and interquartile ranges (Brorsson et al., 2019; Ibrahim et al., 2021; Steinbeck et al., 2015; Wong et al., 2017) were converted to means and standard deviations following the guidelines in the Cochrane Handbook for Systematic Reviews (Higgins et al., 2022) and previous study (Luo et al., 2018; Wan et al., 2014). Data were extracted from the graphs (Tuomaala et al., 2021) using Adobe Acrobat Reader (Adobe, CA, USA). Eight corresponding authors were contacted to request mean and standard deviation data for values that could not be extracted from graphs or were otherwise missing.

### Quality assessment

The quality of the selected articles was independently assessed using the Cochrane risk of bias tool for randomized trials (RoB 2) (Higgins et al., 2022). Researchers 1 and 2 independently assessed the risk of bias for each study. Any discrepancies between researchers 1 and 2 were resolved by researcher 3.

### Data analysis

Meta‐analyses (quantitative synthesis) were performed using the studies with outcome measures. The mean and standard deviation of the pre‐intervention and >6‐month follow‐up results were used to determine the long‐term effects of the intervention. The effect size and heterogeneity of the interventions in the selected studies were analyzed using the RevMan 5.3 version of the Cochrane Collaboration. Publication bias and sensitivity analyses were performed using R Studio 2022.12.0. A random‐effects model was employed for the meta‐analysis due to the variability in interventions among the included studies. For continuous outcome variables, means and standard deviations were presented. When outcome variables were measured with different instruments, effect sizes were calculated using the standardized mean difference (SMD). The heterogeneity of the included studies was assessed using Higgins *I* ^2^ statistics, where 0%, 30%–60%, and 75% of *I* ^2^ indicated no, moderate, and high heterogeneity, respectively (Deeks et al., 2019). Subgroup analysis was performed to evaluate heterogeneity and compare the effects across different subgroups. Studies involving multiple groups were consolidated into single pairwise comparisons (Higgins et al., 2022). Sensitivity analysis was conducted using the leave‐one‐out method, and the Baujat plots were examined to detect outliers among the studies. Finally, publication bias was evaluated using funnel plot and Egger's test to verify the validity of the study (Egger et al., 1997).

## RESULTS

### Characteristics of included studies

**FIGURE 1:**
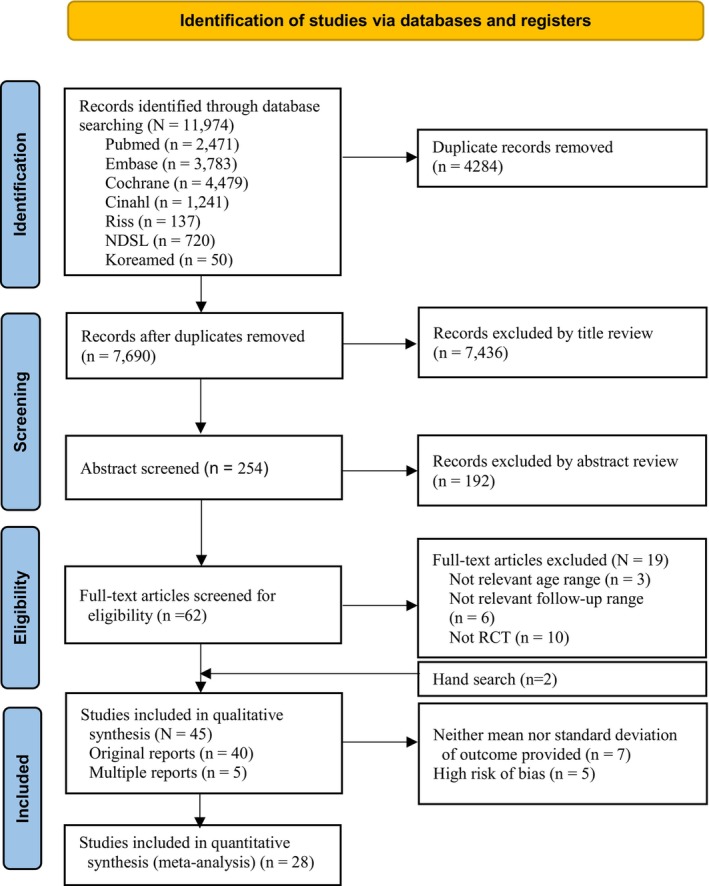
Flow diagram of study selection process.

The search process is illustrated in Figure 1. After excluding 4284 duplicates from 11,974 records, 7690 studies were remained. Additionally, 7436 studies were excluded after reviewing the titles based on the inclusion and exclusion criteria. Subsequently, the abstracts of 254 studies were reviewed, and 192 studies were further excluded, leaving 62 studies for primary screening. Researchers 1 and 2 reviewed the full text of these 62 studies for eligibility. Nineteen studies were excluded: three did not meet age criteria, six had inadequate follow‐up periods, and 10 did not meet the specified study design. Ultimately, 43 studies met the criteria, and two additional studies were found through manual searches. Among these, nine studies (reported as two, three, two, and two) were identified as duplicates or multiple reports and were thus treated as a single study in the meta‐analysis, resulting in a total of 40 studies.

Eight of the included studies did not report the statistical values required for the meta‐analysis. Authors were contacted to obtain data and only one author (Tuomaala) referred a manuscript that values could be found from the tables and figures. Values were extracted from the graphs using the measurement tool in Adobe Acrobat Reader. Ultimately, 40 studies were included in the qualitative synthesis. For the quantitative synthesis, an additional 5 studies were excluded due to a high risk of bias, leaving 28 studies for quantitative analysis (Appendix S3).

### Risk of bias

The risk of bias assessment for each study is presented in Appendix S4. Forty studies were evaluated using RoB 2. Eighteen studies had some concerns regarding the randomization process either due to the absence of allocation concealment or uncertainty about its implementation. For bias arising from deviations in interventions, 38 studies were rated as low risk, as no deviations from the intended intervention were observed within the trial context. In nine studies, there was some concern about bias due to missing outcome data, primarily because of participants' health status or insufficient information regarding loss to follow‐up. Additionally, blinding of participants was not feasible due to the nature of non‐pharmacological interventions, and observer reporting may have influenced the results since outcome variables were measured using self‐reported instruments. Therefore, outcome measurement bias was a concern in 28 studies. Bias in the reported results was rated as low in 38 studies. Overall, five studies (12.5%) were identified as having a high risk of bias, while 31 studies (77.5%) exhibited some concerns regarding the overall risk of bias.

### Study characteristics

Table 1 summarizes the characteristics of the included studies. The qualitative systematic review included 40 RCTs, of which 28 were eligible for the meta‐analysis based on the availability of extractable outcome variables. Of the 40 studies, 22 (55.0%) were published between 2010 and 2019. The majority were conducted in North America (23 studies, 57.5%) and Europe (14 studies, 35.0%). Among these, 22 studies (55.0%) included Whites participants, and 13 studies (32.5%) included African Americans participants. A combination of self‐care behavior management, education, and psychological interventions (11 studies, 27.5%) was the most common type of intervention. Further, 18 studies (45.0%) had follow‐up periods of 7–12 months. Intervention delivery formats varied, with face‐to‐face interventions being the most common (17 studies, 42.5%). Further details are provided in Table S1.

**TABLE 1:** Summary of baseline characteristics for non‐pharmacological studies.

Characteristics	Categories	*N* = 40^a^	*N* = 28^b^
		*n* (%)	*n* (%)
Publication year	<2010	8 (20.0)	7 (25.0)
	2010–2019	22 (55.0)	15 (53.6)
	≥2020	10 (25.0)	6 (21.4)
Publication country	Africa	1 (2.5)	1 (3.6)
	Australia	1 (2.5)	1 (3.6)
	Egypt	1 (2.5)	1 (3.6)
	Europe	14 (35.0)	13 (46.3)
	North America	23 (57.5)	12 (42.9)
Sample size	<100	29 (72.5)	21 (75.0)
	100–199	6 (15.0)	4 (14.3)
	≥200	5 (12.5)	3 (10.7)
Mean age of participants	<12	1 (2.5)	1 (3.6)
	12–17	35 (87.5)	25 (89.3)
	≥18	3 (7.5)	2 (7.1)
	NR	1 (2.5)	0 (0.0)
Race/ethnicity	African American	13 (32.5)	8 (28.6)
	Asian	4 (10.0)	2 (7.1)
	Hispanic	6 (15.0)	3 (10.7)
	White	22 (55.0)	11 (39.3)
	Other^c^	19 (47.5)	19 (67.9)
	NR	17 (42.5)	15 (53.6)
Duration of diabetes diagnosis (year)	4–6	18 (45.0)	12 (42.9)
	≥7	14 (35.0)	11 (39.3)
	NR	8 (20.0)	5 (17.9)
Intervention type	Self‐care behavior management (A)	9 (22.5)	6 (21.4)
	(A) + education (B)	7 (17.5)	6 (21.4)
	(A) + (B) + psychological interventions (C)	11 (27.5)	7 (25.0)
	(A) + (C)	10 (25.0)	6 (21.4)
	(B) + (C)	3 (7.5)	3 (10.7)
Intervention duration (months)	<7	20 (50.0)	12 (42.9)
(months)	7–12	16 (40.0)	13 (46.4)
	≥13	4 (10.0)	3 (10.7)
Intervention frequency	1–6 time/week	11 (27.5)	7 (25.0)
	1–3 time/month	8 (20.0)	6 (21.4)
	Daily	7 (17.5)	3 (10.7)
	NR	5 (12.5)	4 (14.3)
	Others^d^	9 (22.5)	8 (28.6)
Follow‐up duration (months)	6	17 (42.5)	11 (39.3)
	7–12	18 (45.0)	13 (46.4)
	≥13	5 (12.5)	4 (14.3)
Format	App or web (A)	6 (15.0)	3 (10.7)
	Face‐to‐face (B)	17 (42.5)	13 (46.4)
	Telephone (C)	5 (12.5)	3 (10.7)
	(A) + (B)	2 (5.0)	1 (3.6)
	(B) + (C)	7 (17.5)	6 (21.4)
	(A) + (B) + (C)	3 (7.5)	2 (7.1)

Abbreviation: NR, not reported.

^a^ Studies met inclusion criteria.

^b^ Studies included in meta‐analysis.

^c^ Afghanistan, Asian or Pacific Islander, biracial and Hispanic, Danish, ethnic minority, France, Hispanic or Latino, Morocco, multiracial, non‐White or Hispanic, other ethnic or racial background, other non‐Hispanic, Pakistan, Poland, Russia, Somalia, Sweden, Tunisia, Turkey, unknown.

^d^ Once every 4 months, once every 3 months, 8 times in 18 months, and 4 times in 9 months.

Of the 28 studies included in the meta‐analysis, 15 (53.6%) were published between 2010 and 2019. The majority were conducted in Europe (13 studies, 46.3%) and North America (12 studies, 42.9%). Among these, 11 studies (39.3%) included Whites participants, and 8 studies (28.6%) included African Americans participants. The types of interventions varied, with a combination of self‐care behavior management, education, and psychological interventions being the most common (7 studies, 25.0%). Intervention durations ranged from seven to 12 months in 13 studies (46.4%), with six studies (21.4%) providing sessions one to three times per month. Additionally, 13 studies (46.4%) had a follow‐up period of seven to 12 months. Face‐to‐face was the most common intervention delivery format (13 studies, 46.4%).

### Types of interventions for diabetes management: Self‐care, education, and psychological interventions

The 40 studies were classified according to the characteristics of the interventions: self‐care behavioral management, education, and psychological interventions. Self‐care behavior management encompasses activities such as problem‐solving for challenges in diabetes management, blood glucose monitoring, physical activity, carbohydrate counting, goal setting, and dietary control (de Abreu de Lima et al., 2022; Hou et al., 2016; Pillay et al., 2015). Education aims to provide necessary knowledge for diabetes management through quizzes and pamphlets and to support patients in gaining confidence in diabetes self‐care (Charalampopoulos et al., 2017). Psychological interventions focus on improving communication skills between patients and healthcare providers, conducting motivational interviews (MI), stress management, reflection worksheets, and peer support (Channon et al., 2007; Dong et al., 2023; Winkley et al., 2020).

#### Effects of non‐pharmacological interventions on HbA1c

Of the 28 studies included in the meta‐analysis, 19 studies examined the effects of non‐pharmacological interventions on HbA1c levels at the 6‐month follow‐up (Table 2), showing effect size of −0.33 (95% CI: −0.59, −0.06, *p* = .020) with high heterogeneity (*I* ^2^ = 84.0%). Fifteen studies reported the effects of non‐pharmacological interventions on HbA1c levels at follow‐up periods of 7–12 months, and five studies reported follow‐up periods of >12 months, both showing non‐significant effect sizes.

**TABLE 2:** Effect of non‐pharmacologic interventions over follow‐up duration (*N* = 28).

Outcomes	6 months			7–12 months			>12 months		
	*k* ^a^	SMD (95% CI, *p*)	*I* ^2^ (*p*)	*k* ^a^	SMD (95% CI, *p*)	*I* ^2^ (*p*)	*k* ^a^	SMD (95% CI, *p*)	*I* ^2^ (*p*)
Positive affective response^b^	4	0.14 (−0.08, 0.36, .210)	0.0 (.490)	2	0.14 (−0.43, 0.72, .620)	66.0 (.090)	–	–	–
Negative affective response^c^	2	−0.34 (−0.79, 0.11, .140)	0.0 (.530)	4	−0.08 (−0.42, 0.26, .640)	41.0 (.170)	3	−0.10 (−0.38, 0.18, .470)	62.0 (.110)
BMI	2	0.04 (−1.52, 1.60, .960)	95.0 (<.001)	–	–	–	–	–	–
Family conflict	2	−0.18 (−0.53, 0.18, .330)	0.0 (.520)	2	−0.03 (−0.44, 0.38, .890)	25.0 (.250)	–	–	–
Frequency of blood glucose monitoring	6	0.35 (−0.10, 0.80, .120)	78.0 (<.001)	2	0.37 (−0.71, 1.45, .500)	74.0 (.050)	–	–	–
HbA1c	19	−0.33 (−0.59, −0.06, .020)	84.0 (<.001)	15	−0.09 (−0.31, 0.12, .400)	69.0 (<.001)	5	0.01 (−0.13, 0.14, .910)	0.0 (.580)
Insulin dose	2	−0.93 (−2.82, 0.96, .340)	97.0 (<.001)	–	–	–	–	–	–
Quality of life	2	0.13 (−0.15, 0.42, .360)	0.0 (.530)	2	−0.49 (−2.21, 1.23, .580)	93.0 (<.001)	–	–	–
Self‐care	–	–	–	2	0.20 (−0.09, 0.50, .180)	14.0 (.280)	2	0.23 (0.06, 0.40, .009)	0.0 (.670)
Self‐efficacy	2	0.00 (−0.42, 0.43, .980)	0.0 (.790)	3	0.12 (−0.25, 0.48, .530)	0.0 (.960)	–	–	–

Abbreviations: BMI, body mass index; CI, confidence interval; SMD, standardized mean difference.

^a^ Number of studies.

^b^ Affective response, satisfaction.

^c^ Depression, distress, stress.

#### Effects of non‐pharmacological interventions on self‐care

Of the 28 studies included in the meta‐analysis, two studies reported the effects of non‐pharmacological interventions on self‐care at >12‐month follow‐up, showing effect size of 0.23 (95% CI: 0.06, 0.40, *p* = .009) with no heterogeneity (*I* ^2^ = 0.0%).

#### Effects of non‐pharmacological interventions on other outcomes

There were no significant effects of non‐pharmacological interventions on positive affective response, negative affective response, body mass index (BMI), family conflict, frequency of blood glucose monitoring, insulin dose, quality of life, or self‐efficacy at follow‐up periods of 6 months, 7–12 months, and >12 months (Table 2).

#### Subgroup analysis

Subgroup analyses were conducted by classifying the intervention types and follow‐up duration (Table 3). Examining the effects of an intervention type and follow‐up periods of >12 months revealed that an intervention combining self‐care behavior management, education, and psychological interventions significantly improved self‐care, showing effect size of 0.23 (95% CI: 0.06, 0.40, *p* = .009) with no heterogeneity (*I* ^2^ = 0.0%).

**TABLE 3:** Subgroup analysis for the effects of follow‐up duration by type of non‐pharmacologic intervention (*N* = 28).

Intervention type	Outcomes	6 months			7–12 months			>12 months		
		*k* ^a^	SMD (95% CI, *p*)	*I* ^2^ (*p*)	*k* ^a^	SMD (95% CI, *p*)	*I* ^2^ (*p*)	*k* ^a^	SMD (95% CI, *p*)	*I* ^2^ (*p*)
Self‐care behavior management	Positive affective response^b^	2	0.26 (−0.07, 0.58, .130)	0.0 (.540)	–	–	–	–	–	–
	HbA1c	4	−0.69 (−1.53, 0.16, .110)	94.0 (<.001)	3	−0.51 (−1.28, 0.26, .190)	84.0 (.002)	–	–	–
Self‐care behavior management + education	HbA1c	2	−0.35 (−0.74, 0.03, .070)	0.0 (.420)	3	0.09 (−0.30, 0.49, .650)	46.0 (.160)	2	0.05 (−0.20, 0.29, .720)	0.0 (.650)
Self‐care behavior management + education + psychological intervention	Negative affective response^c^	–	–	–	–	–	–	2	−0.10 (−0.38, 0.18, .470)	62.0 (.110)
	Frequency of blood glucose monitoring	2	0.12 (−0.28, 0.53, .550)	50.0 (.160)	–	–	–	–	–	–
	HbA1c	6	−0.15 (−0.30, 0.00, .060)	0.0 (.650)	4	−0.13 (−0.36, 0.09, .250)	42.0 (.160)	2	0.02 (−0.15, 0.19, .800)	0.0 (.350)
	Quality of life	2	0.13 (−0.15, 0.42, .360)	0.0 (.530)	–	–	–	–	–	–
	Self‐care	–	–	–	–	–	–	2	0.23 (0.06, 0.40, .009)	0.0% (.670)
Self‐care behavior management + psychological intervention	Frequency of blood glucose monitoring	3	0.17 (−0.39, 0.72, .560)	49.0 (.140)	–	–	–	–	–	–
	HbA1c	5	−0.41 (−1.14, 0.32, .270)	87.0 (<.001)	3	−0.06 (−0.68, 0.57, .860)	57.0 (.100)	–	–	–
Education + psychological intervention	HbA1c	2	0.19 (−0.50, 0.89, .580)	71.0 (.060)	2	0.40 (−0.06, 0.85, .090)	35.0 (.220)	–	–	–

Abbreviations: CI, confidence interval; SMD, standardized mean difference.

^a^ Number of studies.

^b^ Affective response, satisfaction.

^c^ Depression, distress, stress.

Subgroup analyses examining the effects of intervention format, frequency, and duration (Table 4) showed improvements in HbA1c over the 6‐month follow‐up for interventions combining self‐care behavioral management, education, and psychological interventions, as well as those delivered through face‐to‐face and telephone approaches, with an effect size of −0.24 (95% CI: −0.43, −0.04, *p* = .020). No significant effects of intervention frequency and duration were observed on HbA1c improvement.

**TABLE 4:** Subgroup analysis for the effects of intervention format, duration and frequency on HbA1c levels (*N* = 28).

Intervention type^b^		6 months			7–12 months			>12 months		
		*k* ^a^	SMD (95% CI, *p*)	*I* ^2^ (*p*)	*k* ^a^	SMD (95% CI, *p*)	*I* ^2^ (*p*)	*k* ^a^	SMD (95% CI, *p*)	*I* ^2^ (*p*)
Intervention format										
Face‐to‐face	Total	10	−0.44 (−0.92, .0.03, .070)	89.0 (<.001)	8	−0.09 (−0.31, 0.13, .440)	37.0 (.130)	2	−0.06 (−0.51, 0.39, .790)	55.0 (.140)
	(A) + (B) + (C)	2	−0.10 (−0.47, 0.27, .600)	0.0 (.510)	3	−0.13 (−0.47, 0.21, .460)	51.0 (.130)	–	–	–
	(A) + (C)	5	−0.41 (−1.14, 0.32, .270)	87.0 (<.001)	3	−0.06 (−0.68, 0.57, .860)	57.0 (.100)	2	−0.06 (−0.51, 0.39, .790)	55.0 (.140)
Face‐to‐face+phone	Total	4	−0.12 (−0.42, 0.19, .470)	55.0 (.080)	4	0.00 (−0.35, 0.36, .980)	58.0 (.070)	–	–	–
	(A)	–	–	–	2	−0.14 (−0.52, 0.24, .470)	0.0 (.820)	–	–	–
	(A) + (B) + (C)	3	−0.24 (−0.43, −0.04, .020)	0.0 (.880)	–	–	–	–	–	–
Phone	Total	2	−0.23 (−0.75, 0.30, .400)	54.0 (.140)	–	–	–	–	–	–
Intervention frequency										
Daily	Total	2	−0.50 (−1.50, 0.49, .320)	90.0 (.001)	2	−0.54 (−1.88, 0.80, .430)	95.0 (<.001)	–	–	–
	(A)	2	−0.50 (−1.50, 0.49, .320)	90.0 (.001)	–	–	–	–	–	–
≥1 time/week	Total	7	−0.44 (−0.97, 0.09, .100)	89.0 (<.001)	–	–	–	–	–	–
	(A) + (B)	2	−0.35 (−0.74, 0.03, .070)	0.0 (.420)	–	–	–	–	–	–
	(A) + (B) + (C)	2	−0.06 (−0.39, 0.26, .710)	32.0 (.220)	–	–	–	–	–	–
1–3 time/month	Total	4	−0.12 (−0.39, 0.16, .400)	33.0 (.210)	5	−0.13 (−0.32, 0.06, .170)	24.0 (.260)	2	0.02 (−0.15, 0.19, .800)	0.0 (.350)
	(A) + (B) + (C)	3	−0.19 (−0.39, 0.01, .060)	0.0 (.600)	3	−0.15 (−0.31, 0.01, .060)	0.0 (.460)	2	0.02 (−0.15, 0.19, .800)	0.0 (.350)
Intervention duration										
<7 months		3	−0.83 (−1.80, 0.13, .090)	93.0 (<.001)	–	–	–	–	–	–
7–12 months		7	−0.37 (−0.97, 0.23, .230)	87.0 (<.001)	12	−0.07 (−0.37, 0.24, .670)	73.0 (<.001)	–	–	–
≥13 months		–	–	–	–	–	–	3	−0.01 (−0.18, 0.16, .930)	0.0 (.750)

Abbreviations: CI, confidence interval; SMD, standardized mean difference.

^a^ Number of studies.

^b^ (A) = self‐care behavior management, (B) = education, (C) = psychological management.

### Publication bias

Funnel plots (Appendix S5) and Egger's linear regression tests (*p* = .677) revealed no publication bias for HbA1c. Other outcome variables were not analyzed for publication bias because 10 or fewer studies were included in the analysis.

### Sensitivity analysis

A leave‐one‐out approach was employed to conduct a sensitivity analysis of HbA1c, which involved performing a meta‐analysis on each subset of studies obtained by excluding exactly one study (Higgins et al., 2022) (Appendix S6–S8).

A random‐effects model at 6‐month follow‐up for HbA1c revealed an overall pooled SMD of −0.33 (95% CI: −0.59, −0.06) and heterogeneity of *I* ^2^ = 84.3%. When each study was excluded, the overall SMD and heterogeneity varied from −0.37 to −0.25 and 71.1% to 85.2%, respectively. The study conducted by Salem et al. (2010) was the most influential on the pooled SMDs and heterogeneity levels, according to a Baujat plot. When the study was excluded, the pooled SMD decreased to − 0.25 (95% CI: −0.45, −0.04), and the heterogeneity levels decreased to 71.1%.

A random‐effects model at 7–12 months follow‐up for HbA1c revealed an overall pooled SMD of −0.09 (95% CI: −0.31, 0.12) and heterogeneity of *I* ^2^ = 69.1%. When each study was excluded, the overall SMD and heterogeneity varied from −0.14 to −0.02 and 41.5% to 71.3%, respectively. The study conducted by Chatzakis et al. (2019) was the most influential on the pooled SMDs and heterogeneity levels, according to a Baujat plot. When the study was excluded, the pooled SMD decreased to −0.02 (95% CI: −0.18, 0.14).

A random‐effects model at >12‐month follow‐up for HbA1c revealed an overall pooled SMD of 0.01 (95% CI: −0.13, 0.14) and heterogeneity of *I* ^2^ = 0.0%. When each study was excluded, the overall SMD varied from −0.04 to 0.04. However, heterogeneity did not vary. The study conducted by Hood et al. (2018) exhibited the greatest influence on the pooled SMDs.

## DISCUSSION

This study evaluated the long‐term effects of non‐pharmacological interventions through a systematic literature review and meta‐analysis. A total of 40 RCT studies were included in the systematic review, of which 28 were included in the meta‐analysis. In terms of long‐term effectiveness, non‐pharmacological interventions were effective in improving HbA1c at 6‐month follow‐up, whereas self‐care was effective at >12 months. For other outcome variables, such as lipid levels and BMI, the number of included studies ranged from two to four. As a result, lipid levels and BMI were not analyzed. Additionally, the participant composition was predominantly White, followed by African American, with fewer studies including Asians and other racial groups. This homogeneity limits generalizability of the results to more diverse populations.

The incidence of complications in adolescents increases with the duration of diabetes, and HbA1c levels at the time of T1D diagnosis influence HbA1c levels 10 years later, highlighting the need for long‐term interventions in diabetes management (Mazarello Paes et al., 2019). Adherence to recommended health guidelines is essential for effective diabetes management (Sabbah et al., 2024). Compared with school‐aged children, adolescents with T1D show lower adherence to diabetes management (Sabbah et al., 2024), possibly due to factors such as academic pressure, puberty, reduced parental support, and the overall challenges of managing diabetes (Carlsund & Söderberg, 2018; Saylor et al., 2019). Therefore, long‐term follow‐up studies on non‐pharmacological interventions aimed at reducing diabetic complications in adolescents and young adults with persistent T1D are imperative.

Our results revealed that non‐pharmacological interventions improved HbA1c levels in adolescents and young adults with T1D at 6 months of follow‐up. These results are consistent with those of the meta‐analyses examining the long‐term effects of mobile app interventions (Wu et al., 2019), behavioral programs (Pillay et al., 2015), and cognitive behavioral therapy (CBT) (Dong et al., 2023). Patients with T1D must engage in lifelong self‐management to achieve glycemic control goals and prevent complications, which presents a significant challenge in T1D management. In a subgroup analysis of three studies that showed a reduction in HbA1c at a 6‐month follow‐up, the adherence rate ranged from 74.6% to 78.3% (Chatzakis et al., 2019; Husted et al., 2014; Salem et al., 2010). These studies primarily implemented self‐management behavioral interventions. Dong et al. (2023) reported a reduction in HbA1c levels during long‐term follow‐up (>6 months) in patients with T1D following CBT administration. Among the psychological interventions included in this meta‐analysis (Al Ksir et al., 2022; Channon et al., 2007; Fiallo‐Scharer et al., 2019; Hannon et al., 2018; Mayer‐Davis et al., 2018; Pulkkinen et al., 2020; Stanger et al., 2018; Tuomaala et al., 2021; Wang et al., 2010), MI was the most common but was not effective in improving HbA1c levels at 6‐month follow‐up. However, CBT as a psychological intervention has been effective in improving HbA1c levels at longer follow‐ups (>6 months) (Dong et al., 2023; Winkley et al., 2020). CBT is commonly used to treat dysfunctional cognitive beliefs and behaviors. In conclusion, non‐pharmacological interventions may effectively improve HbA1c levels in adolescents and young adults with T1D; however, further research is needed to develop interventions that enhance long‐term outcomes.

The effect of HbA1c reduction was analyzed by intervention type and follow‐up period, revealing statistically significant improvement in self‐care in two studies (Hood et al., 2018; Mayer‐Davis et al., 2018) that utilized a combination of self‐care behavior management, educational and psychological interventions. Similarly, a behavioral program improved diabetes self‐management adherence in adolescents and young adults with T1D at 12‐month follow‐up (Pillay et al., 2015). Notably, Pillay et al. (2015) focused on facilitating problem‐solving skills, enhancing communication skills, and promoting shared responsibility. They also identified barriers to diabetes management and goal setting, while providing diabetes education materials to improve overall diabetes management. Furthermore, Pulkkinen et al. (2020) and Sabbah et al. (2024) implemented psychological interventions through resilience programs and MI to address T1D‐specific challenges. They provided education by reviewing concepts in each session, distributing a workbook containing content to improve diabetes management, and offering diabetes‐related educational materials. In addition, self‐management interventions employed problem‐solving and communication skills, such as negotiation, assertiveness, and decision‐making. Both the present and a previous study (Pillay et al., 2015) demonstrated that the combination of self‐care behavior management, education, and psychological intervention was effective at follow‐ups beyond 12 month. However, analyses on self‐care behavior management at 6 and 7–12 months of follow‐up could not be conducted due to the insufficient number of studies. Previous research has shown that behavioral programs for diabetes management did not significantly improve self‐care within 12 months (Pillay et al., 2015). Further long‐term effectiveness studies of ongoing non‐pharmacological interventions are warranted to enhance self‐care in adolescents and young adults with T1D.

This study conducted a subgroup analysis of non‐pharmacological interventions based on intervention format to assess their impact on HbA1c levels across different follow‐up duration. Significant reduction in HbA1c were observed in three studies that combined face‐to‐face and telephonic communication formats. Hou et al. (2016), in their meta‐analysis involving patients with T1D, reported that the combination of face‐to‐face and telephonic communication significantly reduced HbA1c levels. A previous meta‐analysis of digital interventions also reported significant reductions in HbA1c levels in patients with T1D when using a combination of face‐to‐face and telephonic delivery methods (Wu et al., 2019). Traditional face‐to‐face interventions can effectively improve HbA1 and cardiovascular risk factors (Salem et al., 2010). However, these interventions may be limited in their intervention. Combining face‐to‐face interventions with digital health strategies, such as mobile apps or Internet‐based resources, can enhance their effectiveness (Hou et al., 2016; Wu et al., 2019).

### Limitations

This study had several limitations. First, most studies were conducted in North America and Europe, which limits the generalizability of the findings to other regions. Second, the impact of non‐pharmacological interventions on cardiovascular risk factors (cholesterol, LDL, HDL, and triglycerides) could not be analyzed due to an insufficient number of studies available for review. Future research is needed to investigate the long‐term follow‐up effects of non‐pharmacological interventions on cardiovascular risk factors. Third, many studies did not provide detailed descriptions of their allocation concealment and blinding procedures. Future studies should emphasize these aspects to enhance research quality. Fourth, there was a lack of long‐term follow‐up in the non‐pharmacological interventions compared to studies that assessed pre‐ and post‐intervention values in adolescents with T1D (Lee et al., 2024). Future research is needed to investigate the long‐term follow‐up effects of non‐pharmacological interventions in adolescents with T1D.

### Implications for practice

This study analyzed the long‐term effects of non‐pharmacological interventions in adolescents and young adults with T1D. Improvements in HbA1c at 6 months and enhanced self‐care at follow‐ups of 12 months or longer were identified. Additionally, combined interventions proved to be more effective than single interventions, with those incorporating both face‐to‐face and telephonic interactions showing greater efficacy. However, due to the lack of studies on long‐term effects in the literature, analyzing the impacts on other outcome variables was challenging, highlighting the need for further research.

## LINKING EVIDENCE TO ACTION

- Non‐pharmacological interventions exert positive effects on HbA1c levels and self‐management improvements in adolescents and young adults with T1D during long‐term follow‐up.
- Combined interventions, including those incorporating both face‐to‐face and phone‐based components, exhibit greater effectiveness in improving HbA1c levels than single interventions.
- Further longitudinal studies exploring the long‐term effects of non‐pharmacological interventions are warranted to analyze their sustained impact on variables such as cardiovascular risk factors. These additional investigations could provide a more comprehensive perspectives on the effects of non‐pharmacological interventions in adolescents and young adults with T1D.

## CONCLUSION

This study evaluated the long‐term effects of non‐pharmacological interventions in adolescents and young adults with T1D through a systematic review and meta‐analysis of 40 RCTs. Our findings demonstrated the effectiveness of non‐pharmacological interventions in improving HbA1c at 6 months and self‐care at follow‐ups of 12 months or longer. Combined interventions with face‐to‐face and telephonic communication were more effective than single interventions. However, analyzing the effects on a broader range of outcome variables was challenging due to insufficient number of studies, highlighting the need for long‐term investigation in the future.

## FUNDING INFORMATION

This research was supported by Basic Science Research Program through the National Research Foundation of Korea (NRF) funded by the Ministry of Education (RS‐2023‐00250276).

## CONFLICT OF INTEREST STATEMENT

The authors declare no conflicts of interest.

## REGISTRATION INFORMATION

The protocol for this review is registered in PROSPERO (registration number: CRD42024503185).

## Supporting information


Appendix S1‐S8

Table S1

## Data Availability

Data supporting the findings of this study are available from the corresponding author upon request.

## References

[wvn12751-bib-0001] Al Ksir, K. , Wood, D. L. , Hasni, Y. , Sahli, J. , Quinn, M. , & Ghardallou, M. (2022). Motivational interviewing to improve self‐management in youth with type 1 diabetes: A randomized clinical trial. Journal of Pediatric Nursing, 66, e116–e121. 10.1016/j.pedn.2022.05.001 35568602

[wvn12751-bib-0002] Brorsson, A. L. , Leksell, J. , Andersson Franko, M. , & Lindholm Olinder, A. (2019). A person‐centered education for adolescents with type 1 diabetes‐a randomized controlled trial. Pediatric Diabetes, 20(7), 986–996. 10.1111/pedi.12888 31268224

[wvn12751-bib-0003] Carlsund, Å. , & Söderberg, S. (2018). Living with type 1 diabetes as experienced by young adults. Nursing Open, 6(2), 418–425. 10.1002/nop2.222 30918692 PMC6419143

[wvn12751-bib-0004] Channon, S. J. , Huws‐Thomas, M. V. , Rollnick, S. , Hood, K. , Cannings‐John, R. L. , Rogers, C. , & Gregory, J. W. (2007). A multicenter randomized controlled trial of motivational interviewing in teenagers with diabetes. Diabetes Care, 30(6), 1390–1395. 10.2337/dc06-2260 17351283

[wvn12751-bib-0005] Charalampopoulos, D. , Hesketh, K. R. , Amin, R. , Paes, V. M. , Viner, R. M. , & Stephenson, T. (2017). Psycho‐educational interventions for children and young people with type 1 diabetes in the UK: How effective are they? A systematic review and meta‐analysis. PLoS One, 12(6), e0179685. 10.1371/journal.pone.0179685 28665946 PMC5493302

[wvn12751-bib-0006] Chatzakis, C. , Floros, D. , Papagianni, M. , Tsiroukidou, K. , Kosta, K. , Vamvakis, A. , Koletsos, N. , Hatziagorou, E. , Tsanakas, I. , & Mastorakos, G. (2019). The beneficial effect of the mobile application e*uglyca* in children and adolescents with type 1 diabetes mellitus: A randomized controlled trial. Diabetes Technology & Therapeutics, 21(11), 627–634. 10.1089/dia.2019.0170 31335204

[wvn12751-bib-0007] de Abreu de Lima, V. , de Menezes, F. J., Jr. , da Rocha Celli, L. , França, S. N. , Cordeiro, G. R. , Mascarenhas, L. P. G. , & Leite, N. (2022). Effects of resistance training on the glycemic control of people with type 1 diabetes: A systematic review and meta‐analysis. Archives of Endocrinology Metabolism, 66(4), 533–540. 10.20945/2359-3997000000487 35758833 PMC10697639

[wvn12751-bib-0008] Deeks, J. J. , Higgins, J. P. , Altman, D. G. , & Cochrane Statistical Methods Group . (2019). Analysing data and undertaking meta‐analyses. In Cochrane handbook for systematic reviews of interventions (2nd ed., pp. 241–284). Cochrane. 10.1002/9781119536604.ch10

[wvn12751-bib-0009] Dong, N. , Wang, X. , & Yang, L. (2023). The short‐ and long‐term effects of cognitive behavioral therapy on the glycemic control of diabetic patients: A systematic review and meta‐analysis. BioPsychoSocial Medicine, 17(1), 18. 10.1186/s13030-023-00274-5 37150826 PMC10165773

[wvn12751-bib-0010] Egger, M. , Davey Smith, G. , Schneider, M. , & Minder, C. (1997). Bias in meta‐analysis detected by a simple, graphical test. BMJ, 315(7109), 629–634. 10.1136/bmj.315.7109.629 9310563 PMC2127453

[wvn12751-bib-0011] Fiallo‐Scharer, R. , Palta, M. , Chewning, B. A. , Rajamanickam, V. , Wysocki, T. , Wetterneck, T. B. , & Cox, E. D. (2019). Impact of family‐centered tailoring of pediatric diabetes self‐management resources. Pediatric Diabetes, 20(7), 1016–1024. 10.1111/pedi.12899 31355957 PMC6827338

[wvn12751-bib-0012] Hannon, T. S. , Yazel‐Smith, L. G. , Hatton, A. S. , Stanton, J. L. , Moser, E. A. S. , Li, X. , & Carroll, A. E. (2018). Advancing diabetes management in adolescents: Comparative effectiveness of mobile self‐monitoring blood glucose technology and family‐centered goal setting. Pediatric Diabetes, 19(4), 776–781. 10.1111/pedi.12648 29504207 PMC6476179

[wvn12751-bib-0013] Higgins, J. P. T. , Thomas, J. , Chandler, J. , Cumpston, M. , Li, T. , Page, M. J. , & Welch, V. A. (2022). Cochrane handbook for systematic reviews of interventions (version 6.4). Cochrane. https://training.cochrane.org/handbook

[wvn12751-bib-0014] Holman, N. , Woch, E. , Dayan, C. , Warner, J. , Robinson, H. , Young, B. , & Elliott, J. (2023). National trends in hyperglycemia and diabetic ketoacidosis in children, adolescents, and young adults with type 1 diabetes: A challenge due to age or stage of development, or is new thinking about service provision needed? Diabetes Care, 46(7), 1404–1408. 10.2337/dc23-0180 37216620 PMC10300515

[wvn12751-bib-0015] Hood, K. K. , Iturralde, E. , Rausch, J. , & Weissberg‐Benchell, J. (2018). Preventing diabetes distress in adolescents with type 1 diabetes: Results 1 year after participation in the steps program. Diabetes Care, 41(8), 1623–1630. 10.2337/dc17-2556 29921624 PMC6054495

[wvn12751-bib-0016] Hou, C. , Carter, B. , Hewitt, J. , Francisa, T. , & Mayor, S. (2016). Do mobile phone applications improve glycemic control (HbA1c) in the self‐management of diabetes? A systematic review, meta‐analysis, and GRADE of 14 randomized trials. Diabetes Care, 39(11), 2089–2095. 10.2337/dc16-0346 27926892

[wvn12751-bib-0017] Husted, G. R. , Thorsteinsson, B. , Esbensen, B. A. , Gluud, C. , Winkel, P. , Hommel, E. , & Zoffmann, V. (2014). Effect of guided self‐determination youth intervention integrated into outpatient visits versus treatment as usual on glycemic control and life skills: A randomized clinical trial in adolescents with type 1 diabetes. Trials, 15, 321. 10.1186/1745-6215-15-321 25118146 PMC4247629

[wvn12751-bib-0018] Ibrahim, N. , Treluyer, J. M. , Briand, N. , Godot, C. , Polak, M. , & Beltrand, J. (2021). Text message reminders for adolescents with poorly controlled type 1 diabetes: A randomized controlled trial. PLoS One, 16(3), e0248549. 10.1371/journal.pone.0248549 33720997 PMC7959392

[wvn12751-bib-0019] Lee, D. , Lee, H. , Shin, Y. , & Park, G. (2024). Effectiveness of non‐pharmacological interventions for adolescents with type 1 diabetes in the last five years: A systematic review and meta‐analysis. Asian Nursing Research, 18(1), 51–59. 10.1016/j.anr.2024.01.008 38307162

[wvn12751-bib-0020] Liu, F. , Guan, Y. , Li, X. , Xie, Y. , He, J. , Zhou, Z. G. , & Li, L. (2020). Different effects of structured education on glycemic control and psychological outcomes in adolescent and adult patients with type 1 diabetes: A systematic review and meta‐analysis. International Journal of Endocrinology, 2020, 9796019. 10.1155/2020/9796019 32184823 PMC7061135

[wvn12751-bib-0021] Luo, D. , Wan, X. , Liu, J. , & Tong, T. (2018). Optimally estimating the sample mean from the sample size, median, mid‐range, and/or mid‐quartile range. Statistical Methods in Medical Research, 27(6), 1785–1805. 10.1177/0962280216669183 27683581

[wvn12751-bib-0022] Mayer‐Davis, E. J. , Maahs, D. M. , Seid, M. , Crandell, J. , Bishop, F. K. , Driscoll, K. A. , Hunter, C. M. , Kichler, J. C. , Standiford, D. , Thomas, J. M. , & FLEX Study Group . (2018). Efficacy of the flexible lifestyles empowering change intervention on metabolic and psychosocial outcomes in adolescents with type 1 diabetes (FLEX): A randomised controlled trial. The Lancet. Child & Adolescent Health, 2(9), 635–646. 10.1016/S2352-4642(18)30208-6 30119757 PMC6260973

[wvn12751-bib-0023] Mazarello Paes, V. , Barrett, J. K. , Taylor‐Robinson, D. C. , Chesters, H. , Charalampopoulos, D. , Dunger, D. B. , Viner, R. M. , & Stephenson, T. J. (2019). Effect of early glycemic control on HbA1c tracking and development of vascular complications after 5 years of childhood onset type 1 diabetes: Systematic review and meta‐analysis. Pediatric Diabetes, 20(5), 494–509. 10.1111/pedi.12850 30932298 PMC6701989

[wvn12751-bib-0024] Muñoz‐Pardeza, J. , López‐Gil, J. F. , Huerta‐Uribe, N. , Hormazábal‐Aguayo, I. , Izquierdo, M. , & García‐Hermoso, A. (2024). Nonpharmacological interventions on glycated haemoglobin in youth with type 1 diabetes: A Bayesian network meta‐analysis. Cardiovascular Diabetology, 23(1), 230. 10.1186/s12933-024-02301-3 38951907 PMC11218128

[wvn12751-bib-0025] Ogle, G. D. , James, S. , Dabelea, D. , Pihoker, C. , Svennson, J. , Maniam, J. , Klatman, E. L. , & Patterson, C. C. (2022). Global estimates of incidence of type 1 diabetes in children and adolescents: Results from the International Diabetes Federation Atlas. Diabetes Research and Clinical Practice, 183, 109083. 10.1016/j.diabres.2021.109083 34883188

[wvn12751-bib-0026] Page, M. J. , Moher, D. , Bossuyt, P. M. , Boutron, I. , Hoffmann, T. C. , Mulrow, C. D. , Shamseer, L. , Tetzlaff, J. M. , Akl, E. A. , Brennan, S. E. , Chou, R. , Glanville, J. , Grimshaw, J. M. , Hróbjartsson, A. , Lalu, M. M. , Li, T. , Loder, E. W. , Mayo‐Wilson, E. , McDonald, S. , … McKenzie, J. E. (2021). PRISMA 2020 explanation and elaboration: Updated guidance and exemplars for reporting systematic reviews. BMJ, 372, n160. 10.1136/bmj.n160 33781993 PMC8005925

[wvn12751-bib-0027] Pillay, J. , Armstrong, M. J. , Butalia, S. , Donovan, L. E. , Sigal, R. J. , Vandermeer, B. , Chordiya, P. , Dhakal, S. , Hartling, L. , Nuspl, M. , Featherstone, R. , & Dryden, D. M. (2015). Behavioral programs for type 1 diabetes mellitus: A systematic review and meta‐analysis. Annals of Internal Medicine, 163(11), 836–847. 10.7326/M15-1399 26414020

[wvn12751-bib-0028] Pulkkinen, M. A. , Tuomaala, A. K. , Hero, M. , Gordin, D. , & Sarkola, T. (2020). Motivational interview to improve vascular health in adolescents with poorly controlled type 1 diabetes (MIAD): A randomized controlled trial. BMJ Open Diabetes Research & Care, 8(1), e001216. 10.1136/bmjdrc-2020-001216 PMC7388880 32723754

[wvn12751-bib-0029] Quirk, H. , Blake, H. , Tennyson, R. , Randell, T. L. , & Glazebrook, C. (2014). Physical activity interventions in children and young people with type 1 diabetes mellitus: A systematic review with meta‐analysis. Diabetic Medicine, 31(10), 1163–1173. 10.1111/dme.12531 24965376 PMC4232875

[wvn12751-bib-0030] Rawshani, A. , Sattar, N. , Franzén, S. , Rawshani, A. , Hattersley, A. T. , Svensson, A. M. , Eliasson, B. , & Gudbjörnsdottir, S. (2018). Excess mortality and cardiovascular disease in young adults with type 1 diabetes in relation to age at onset: A nationwide, register‐based cohort study. Lancet, 392(10146), 477–486. 10.1016/S0140-6736(18)31506-X 30129464 PMC6828554

[wvn12751-bib-0031] Sabbah, M. M. , Hjazeen, A. A. , & Arabiat, D. (2024). Adherence to diabetes management among school‐aged children and adolescents living with type 1 diabetes in Jordan. Journal of Pediatric Nursing, 74, 110–115. 10.1016/j.pedn.2023.11.021 38039929

[wvn12751-bib-0032] Salem, M. A. , AboElAsrar, M. A. , Elbarbary, N. S. , ElHilaly, R. A. , & Refaat, Y. M. (2010). Is exercise a therapeutic tool for improvement of cardiovascular risk factors in adolescents with type 1 diabetes mellitus? A randomised controlled trial. Diabetology & Metabolic Syndrome, 2(1), 47. 10.1186/1758-5996-2-47 20618996 PMC3238209

[wvn12751-bib-0033] Saylor, J. , Hanna, K. M. , & Calamaro, C. J. (2019). Experiences of college students who are newly diagnosed with type 1 diabetes mellitus. Journal of Pediatric Nursing, 44, 74–80. 10.1016/j.pedn.2018.10.020 30683284

[wvn12751-bib-0034] Stanger, C. , Lansing, A. H. , Scherer, E. , Budney, A. , Christiano, A. S. , & Casella, S. J. (2018). A web‐delivered multicomponent intervention for adolescents with poorly controlled type 1 diabetes: A pilot randomized controlled trial. Annals of Behavioral Medicine, 52(12), 1010–1022. 10.1093/abm/kay005 30418521 PMC6230973

[wvn12751-bib-0035] Steinbeck, K. S. , Shrewsbury, V. A. , Harvey, V. , Mikler, K. , Donaghue, K. C. , Craig, M. E. , & Woodhead, H. J. (2015). A pilot randomized controlled trial of a post‐discharge program to support emerging adults with type 1 diabetes mellitus transition from pediatric to adult care. Pediatric Diabetes, 16(8), 634–639. 10.1111/pedi.12229 25385685

[wvn12751-bib-0036] Tuomaala, A. K. , Hero, M. , Tuomisto, M. T. , Lähteenmäki, M. , Miettinen, P. J. , Laine, T. , Wehkalampi, K. , Kiiveri, S. , Ahonen, P. , Ojaniemi, M. , Kaunisto, K. , Tossavainen, P. , Lapatto, R. , Sarkola, T. , & Pulkkinen, M. A. (2021). Motivational interviewing and glycemic control in adolescents with poorly controlled type 1 diabetes: A randomized controlled pilot trial. Frontiers in Endocrinology, 12(639), 507. 10.3389/fendo.2021.639507 PMC7994365 33776935

[wvn12751-bib-0037] Viana, L. V. , Gomes, M. B. , Zajdenverg, L. , Pavin, E. J. , Azevedo, M. J. , & Brazilian Type 1 Diabetes Study Group . (2016). Interventions to improve patients' compliance with therapies aimed at lowering glycated hemoglobin (HbA1c) in type 1 diabetes: Systematic review and meta‐analyses of randomized controlled clinical trials of psychological, telecare, and educational interventions. Trials, 17, 94. 10.1186/s13063-016-1207-6 26888087 PMC4758163

[wvn12751-bib-0038] Wan, X. , Wang, W. , Liu, J. , & Tong, T. (2014). Estimating the sample mean and standard deviation from the sample size, median, range and/or interquartile range. BMC Medical Research Methodology, 14, 135. 10.1186/1471-2288-14-135 25524443 PMC4383202

[wvn12751-bib-0039] Wang, Y. C. , Stewart, S. M. , Mackenzie, M. , Nakonezny, P. A. , Edwards, D. , & White, P. C. (2010). A randomized controlled trial comparing motivational interviewing in education to structured diabetes education in teens with type 1 diabetes. Diabetes Care, 33(8), 1741–1743. 10.2337/dc10-0019 20484124 PMC2909053

[wvn12751-bib-0040] Winkley, K. , Upsher, R. , Stahl, D. , Pollard, D. , Kasera, A. , Brennan, A. , Heller, S. , & Ismail, K. (2020). Psychological interventions to improve self‐management of type 1 and type 2 diabetes: A systematic review. Health Technology Assessment, 24(28), 1–232. 10.3310/hta24280 PMC7336224 32568666

[wvn12751-bib-0041] Wong, C. A. , Miller, V. A. , Murphy, K. , Small, D. , Ford, C. A. , Willi, S. M. , Feingold, J. , Morris, A. , Ha, Y. P. , Zhu, J. , Wang, W. , & Patel, M. S. (2017). Effect of financial incentives on glucose monitoring adherence and glycemic control among adolescents and young adults with type 1 diabetes: A randomized clinical trial. JAMA Pediatrics, 171(12), 1176–1183. 10.1001/jamapediatrics.2017.3233 29059263 PMC6583649

[wvn12751-bib-0042] Wu, X. , Guo, X. , & Zhang, Z. (2019). The efficacy of mobile phone apps for lifestyle modification in diabetes: Systematic review and meta‐analysis. JMIR mHealth and uHealth, 7(1), e12297. 10.2196/12297 30664494 PMC6350094

[wvn12751-bib-0043] Yetim, A. , Alikaşifoğlu, M. , Baş, F. , Eliaçık, K. , Çığ, G. , Erginöz, E. , Ercan, O. , & Bundak, R. (2018). Glycemic control and health behaviors in adolescents with type 1 diabetes. The Turkish Journal of Pediatrics, 60(3), 244–254. 10.24953/turkjped.2018.03.003 30511536

