# Supplementary material for: Long‐term effects of non‐pharmacological interventions in adolescents and young adults with type 1 diabetes: A systematic review and meta‐analysis
Source: Worldviews Evid Based Nurs. 2024 Nov 4;21(6):665–77. doi: 10.1111/wvn.12751 (PMC11655730; doi:10.1111/wvn.12751)
Supplement: Supplementary file 1 — Appendix S1‐S8 [file WVN-21-665-s001.docx]

**Appendix S1**

*PRISMA 2020 Checklist*

| **Section and Topic** | **Item #** | **Checklist item** | **Location where item is reported** |
| --- | --- | --- | --- |
| **TITLE** | | |  |
| Title | 1 | Identify the report as a systematic review. | Title |
| **ABSTRACT** | | |  |
| Abstract | 2 | See the PRISMA 2020 for Abstracts checklist. | Abstract |
| **INTRODUCTION** | | |  |
| Rationale | 3 | Describe the rationale for the review in the context of existing knowledge. | 1–2 |
| Objectives | 4 | Provide an explicit statement of the objective(s) or question(s) the review addresses. | 2 |
| **METHODS** | | |  |
| Eligibility criteria | 5 | Specify the inclusion and exclusion criteria for the review and how studies were grouped for the syntheses. | 2–3 |
| Information sources | 6 | Specify all databases, registers, websites, organisations, reference lists and other sources searched or consulted to identify studies. Specify the date when each source was last searched or consulted. | 3, Appendix S2 |
| Search strategy | 7 | Present the full search strategies for all databases, registers and websites, including any filters and limits used. | 3, Appendix S1 |
| Selection process | 8 | Specify the methods used to decide whether a study met the inclusion criteria of the review, including how many reviewers screened each record and each report retrieved, whether they worked independently, and if applicable, details of automation tools used in the process. | 3 |
| Data collection process | 9 | Specify the methods used to collect data from reports, including how many reviewers collected data from each report, whether they worked independently, any processes for obtaining or confirming data from study investigators, and if applicable, details of automation tools used in the process. | 3–4 |
| Data items | 10a | List and define all outcomes for which data were sought. Specify whether all results that were compatible with each outcome domain in each study were sought (e.g. for all measures, time points, analyses), and if not, the methods used to decide which results to collect. | 4 |
|  | 10b | List and define all other variables for which data were sought (e.g. participant and intervention characteristics, funding sources). Describe any assumptions made about any missing or unclear information. | 3–4 |
| Study risk of bias assessment | 11 | Specify the methods used to assess risk of bias in the included studies, including details of the tool(s) used, how many reviewers assessed each study and whether they worked independently, and if applicable, details of automation tools used in the process. | 4, Appendix S3 |
| Effect measures | 12 | Specify for each outcome the effect measure(s) (e.g. risk ratio, mean difference) used in the synthesis or presentation of results. | 4–5 |
| Synthesis methods | 13a | Describe the processes used to decide which studies were eligible for each synthesis (e.g. tabulating the study intervention characteristics and comparing against the planned groups for each synthesis (item #5)). | 4–5 |
|  | 13b | Describe any methods required to prepare the data for presentation or synthesis, such as handling of missing summary statistics, or data conversions. | 4–5 |
|  | 13c | Describe any methods used to tabulate or visually display results of individual studies and syntheses. | 4–5 |
|  | 13d | Describe any methods used to synthesize results and provide a rationale for the choice(s). If meta-analysis was performed, describe the model(s), method(s) to identify the presence and extent of statistical heterogeneity, and software package(s) used. | 4–5 |
|  | 13e | Describe any methods used to explore possible causes of heterogeneity among study results (e.g. subgroup analysis, meta-regression). | 4–5 |
|  | 13f | Describe any sensitivity analyses conducted to assess robustness of the synthesized results. | 4–5 |
| Reporting bias assessment | 14 | Describe any methods used to assess risk of bias due to missing results in a synthesis (arising from reporting biases). | 3–5 |
| Certainty assessment | 15 | Describe any methods used to assess certainty (or confidence) in the body of evidence for an outcome. | 4–5 |
| **RESULTS** | | |  |
| Study selection | 16a | Describe the results of the search and selection process, from the number of records identified in the search to the number of studies included in the review, ideally using a flow diagram. | 5–6, Figure 1 |
|  | 16b | Cite studies that might appear to meet the inclusion criteria, but which were excluded, and explain why they were excluded. | 5–6, Figure 1 |
| Study characteristics | 17 | Cite each included study and present its characteristics. | 6 |
| Risk of bias in studies | 18 | Present assessments of risk of bias for each included study. | 6–7 |
| Results of individual studies | 19 | For all outcomes, present, for each study: (a) summary statistics for each group (where appropriate) and (b) an effect estimate and its precision (e.g. confidence/credible interval), ideally using structured tables or plots. | 7–8 |
| Results of syntheses | 20a | For each synthesis, briefly summarise the characteristics and risk of bias among contributing studies. | 6–7 |
|  | 20b | Present results of all statistical syntheses conducted. If meta-analysis was done, present for each the summary estimate and its precision (e.g. confidence/credible interval) and measures of statistical heterogeneity. If comparing groups, describe the direction of the effect. | 7–8 |
|  | 20c | Present results of all investigations of possible causes of heterogeneity among study results. | 7–9 |
|  | 20d | Present results of all sensitivity analyses conducted to assess the robustness of the synthesized results. | 8–9 |
| Reporting biases | 21 | Present assessments of risk of bias due to missing results (arising from reporting biases) for each synthesis assessed. | 8–9, Appendix S5–7 |
| Certainty of evidence | 22 | Present assessments of certainty (or confidence) in the body of evidence for each outcome assessed. | 6–7, Appendix S3 |
| **DISCUSSION** | | |  |
| Discussion | 23a | Provide a general interpretation of the results in the context of other evidence. | 9–10 |
|  | 23b | Discuss any limitations of the evidence included in the review. | 12–13 |
|  | 23c | Discuss any limitations of the review processes used. | 12–13 |
|  | 23d | Discuss implications of the results for practice, policy, and future research. | 13 |
| **OTHER INFORMATION** | | |  |
| Registration and protocol | 24a | Provide registration information for the review, including register name and registration number, or state that the review was not registered. | Abstract, 14 |
|  | 24b | Indicate where the review protocol can be accessed, or state that a protocol was not prepared. | 14 |
|  | 24c | Describe and explain any amendments to information provided at registration or in the protocol. | NA |
| Support | 25 | Describe sources of financial or non-financial support for the review, and the role of the funders or sponsors in the review. | 14 |
| Competing interests | 26 | Declare any competing interests of review authors. | 14 |
| Availability of data, code and other materials | 27 | Report which of the following are publicly available and where they can be found: template data collection forms; data extracted from included studies; data used for all analyses; analytic code; any other materials used in the review. | 14 |

**Appendix S2**

*Search Strategy*

| **PubMed** | **Search No** | **Search query** |
| --- | --- | --- |
|  | #1 | "Diabetes Mellitus, Type 1"[Mesh] |
|  | #2 | (((((("Diabetes Mellitus, Type 1"[TW]) OR ("Type 1 Diabetes"[TW])) OR ("Insulin-Dependent Diabetes Mellitus"[TW])) OR ("Juvenile-Onset Diabetes"[TW])) OR ("1 Insulin-Dependent Diabetes Mellitus"[TW])) OR ("IDDM"[TW])) OR ("T1DM"[TW]) |
|  | #3 | #1 OR #2 |
|  | #4 | "Adolescent"[Mesh] |
|  | #5 | ((("Adolescent*"[TW]) OR ("Teen*"[TW])) OR ("Youth*"[TW])) OR ("Juvenile"[TW]) |
|  | #6 | "Young Adult"[Mesh] |
|  | #7 | "Young*"[TW] |
|  | #8 | #4 OR #5 OR #6 OR #7 |
|  | #9 | #3 AND #8 |
|  | #10 | "Randomized Controlled Trial" [Publication Type] |
|  | #11 | ("controlled clinical trial"[PT]) OR ("randomized"[TIAB]) |
|  | #12 | #10 OR #11 |
|  | #13 | #9 AND #12 |
| **EMBASE** | **Search No** | **Search query** |
|  | #1 | 'insulin dependent diabetes mellitus'/exp |
|  | #2 | 'Diabetes Mellitus, Type I' OR 'insulin-dependent diabetes*' OR 'Type 1 Diabetes*' OR 'diabetes type 1' OR 'diabetes type I' OR 'iddm' OR 'dm 1' OR 'T1DM' OR 'Juvenile-Onset Diabetes' OR 'juvenile diabetes' |
|  | #3 | #1 OR #2 |
|  | #4 | 'adolescent'/exp |
|  | #5 | 'teen*' OR 'adolescent*' OR 'youth*' OR 'juvenile' |
|  | #6 | ‘young adult’/exp |
|  | #7 | 'young*' |
|  | #8 | #4 OR #5 OR #6 OR #7 |
|  | #9 | #3 AND #8 |
|  | #10 | 'controlled clinical trial’/exp |
|  | #11 | ‘RANDOMIZATION’/exp |
|  | #12 | #10 OR #11 |
|  | #13 | #9 AND #12 |
| **Cochrane Library** | **Search No** | **Search query** |
|  | #1 | [mh "Diabetes Mellitus, Type 1"] |
|  | #2 | "Diabetes Mellitus, Type I":ti,ab,kw OR "Diabetes, Type 1":ti,ab,kw OR "Type 1 Diabetes":ti,ab,kw OR "Insulin-Dependent Diabetes Mellitus":ti,ab,kw OR "Diabetes Mellitus, Insulin-Dependent":ti,ab,kw OR "Diabetes Mellitus, Juvenile-Onset":ti,ab,kw OR "IDDM":ti,ab,kw OR "T1DM"ti,ab,kw |
|  | #3 | #1 OR #2 |
|  | #4 | [mh "Young Adult"] |
|  | #5 | Young*:ti,ab,kw |
|  | #6 | [mh "Adolescent"] |
|  | #7 | Adolescent*:ti,ab,kw OR Youth*:ti,ab,kw OR Teen*:ti,ab,kw OR "Juvenile":ti,ab,kw |
|  | #8 | #4 OR #5 OR #6 OR #7 |
|  | #9 | #3 AND #8 |
|  | #10 | Random*:ti,ab,kw |
|  | #11 | #9 AND #10 |
| **CINAHL (EBSCO host)** | **Search No** | **Search query** |
|  | S1 | (MH "Diabetes Mellitus, Type 1") |
|  | S2 | TI("Type 1 Diabetes" OR "Diabetes Mellitus, Type 1" OR "Insulin-Dependent Diabetes Mellitus" OR "Juvenile-Onset Diabetes" OR "IDDM" OR "T1DM") OR AB("Type 1 Diabetes" OR "Diabetes Mellitus, Type 1" OR "Insulin-Dependent Diabetes Mellitus" OR "Juvenile-Onset Diabetes" OR "IDDM" OR "T1DM") OR SU("Type 1 Diabetes" OR "Diabetes Mellitus, Type 1" OR "Insulin-Dependent Diabetes Mellitus" OR "Juvenile-Onset Diabetes" OR "IDDM" OR "T1DM") |
|  | S3 | S2 OR S1 |
|  | S4 | (MH "adolescent") |
|  | S5 | TI("Adolescent*" OR "Teen*" OR "Youth*" OR "Juvenile") OR AB("Adolescent*" OR "Teen*" OR "Youth*" OR "Juvenile") OR SU("Adolescent*" OR "Teen*" OR "Youth*" OR "Juvenile") |
|  | S6 | (MH "Young Adult") |
|  | S7 | TI("Young*" ) OR AB("Young*" ) OR SU("Young*" ) |
|  | S8 | S4 OR S5 OR S6 OR S7 |
|  | S9 | S3 AND S8 |
|  | S10 | (MH "Random Assignment") |
|  | S11 | TI("Random*" OR "allocat*" OR "Assignment*") OR AB("Random*" OR "allocat*" OR "Assignment*") OR SU("Random*" OR "allocat*" OR "Assignment*") |
|  | S12 | (MH "Randomized Controlled Trials") |
|  | S13 | (MH "Clinical Trials") |
|  | S14 | TI(Clinical Trial*) OR AB(Clinical Trial*) OR SU(Clinical Trial*) |
|  | S15 | (MH "Random Sample") |
|  | S16 | S10 OR S11 OR S12 OR S13 OR S14 OR S15 |
|  | S17 | S9 AND S16 |

**Appendix S3**

*List of the Studies Included in the Meta-Analysis*

1. Leksell, J., Toft, E., Rosman, J., Eriksson, J. W., Fischier, J., Lindholm-Olinder, A., Rosenblad, A., & Nerpin, E. (2023). Virtual clinic for young people with type 1 diabetes: A randomised wait-list controlled study. *BMC Endocrine Disorders*, *23*(1), 255. https://doi.org/10.1186/s12902-023-01516-x

2. Ibrahim, N., Treluyer, J. M., Briand, N., Godot, C., Polak, M., & Beltrand, J. (2021). Text message reminders for adolescents with poorly controlled type 1 diabetes: A randomized controlled trial. *PLOS ONE*, *16*(3), e0248549. https://doi.org/10.1371/journal.pone.0248549

3. Halper, J. B., Yazel, L. G., El Mikati, H., Hatton, A., Tully, J., Li, X., Carroll, A. E., & Hannon, T. S. (2022). Patient and parent well-being and satisfaction with diabetes care during a comparative trial of mobile self-monitoring blood glucose technology and family-centered goal setting. *Frontiers in Clinical Diabetes and Healthcare, 3,* 769116. https://doi.org/10.3389/fcdhc.2022.769116

4. Kassai, B., Rabilloud, M., Bernoux, D., Michal, C., Riche, B., Ginhoux, T., Laudy, V., Terral, D., Didier-Wright, C., Maire, V., Dumont, C., Cottancin, G., Plasse, M., Jeannoel, G. P., Khoury, J., Bony, C., Lièvre, M., Drai, J., & Nicolino, M. (2015). Management of adolescents with very poorly controlled type 1 diabetes by nurses: A parallel group randomized controlled trial. *Trials*, *16*, 399. https://doi.org/10.1186/s13063-015-0923-7

5. Salem, M. A., AboElAsrar, M. A., Elbarbary, N. S., ElHilaly, R. A., & Refaat, Y. M. (2010). Is exercise a therapeutic tool for improvement of cardiovascular risk factors in adolescents with type 1 diabetes mellitus? A randomised controlled trial. *Diabetology & Metabolic Syndrome*, *2*(1), 47. https://doi.org/10.1186/1758-5996-2-47

6. Morrissey, E. C., Byrne, M., Casey, B., Casey, D., Gillespie, P., Hobbins, A., Lowry, M., McCarthy, E., Newell, J., Roshan, D., Sharma, S., D1 Now Young Adult Panel, & Dinneen, S. F. (2022). Improving outcomes among young adults with type 1 diabetes: The D1 Now pilot cluster randomised controlled trial. *Pilot Feasibility Study*, *8*(1), 56. https://doi.org/10.1186/s40814-022-00986-5

7. Chatzakis, C., Floros, D., Papagianni, M., Tsiroukidou, K., Kosta, K., Vamvakis, A., Koletsos, N., Hatziagorou, E., Tsanakas, I., & Mastorakos, G. (2019). The beneficial effect of the mobile application euglyca in children and adolescents with type 1 diabetes mellitus: A randomized controlled trial. *Diabetes Technology & Therapeutics, 21*(11), 627–634. https://doi.org/10.1089/dia.2019.0170

8. Goyal, S., Nunn, C. A., Rotondi, M., Couperthwaite, A. B., Reiser, S., Simone, A., Katzman, D. K., Cafazzo, J. A., & Palmert, M. R. (2017). A mobile app for the self-management of type 1 diabetes among adolescents: A randomized controlled trial. *JMIR mHealth and uHealth*, *5*(6), e82. https://doi.org/10.2196/mhealth.7336

9. Wong, C. A., Miller, V. A., Murphy, K., Small, D., Ford, C. A., Willi, S. M., Feingold, J., Morris, A., Ha, Y. P., Zhu, J., Wang, W., & Patel, M. S. (2017). Effect of financial incentives on glucose monitoring adherence and glycemic control among adolescents and young adults with type 1 diabetes: A randomized clinical trial. *JAMA Pediatrics*, *171*(12), 1176–1183. https://doi.org/10.1001/jamapediatrics.2017.3233

10. Castensøe-Seidenfaden, P., Husted, G. R., Jensen, A. K., Hommel, E., Olsen, B., Pedersen-Bjergaard, U., Kensing, F., & Teilmann, G. (2018). Testing a smartphone app (young with diabetes) to improve self-management of diabetes over 12 months: Randomized controlled trial. *JMIR mHealth and uHealth*, *6*(6), e141. https://doi.org/10.2196/mhealth.9487

11. Lawson, M. L., Cohen, N., Richardson, C., Orrbine, E., & Pham, B. (2005). A randomized trial of regular standardized telephone contact by a diabetes nurse educator in adolescents with poor diabetes control. *Pediatric Diabetes*, *6*(1), 32–40. https://doi.org/10.1111/j.1399-543X.2005.00091.x

12. Steinbeck, K. S., Shrewsbury, V. A., Harvey, V., Mikler, K., Donaghue, K. C., Craig, M. E., & Woodhead, H. J. (2015). A pilot randomized controlled trial of a post-discharge program to support emerging adults with type 1 diabetes mellitus transition from pediatric to adult care. *Pediatric Diabetes*, *16*(8), 634–639. https://doi.org/10.1111/pedi.12229

13. Kaushal, T., Katz, L. E. L., Joseph, J., Marowitz, M., Morales, K. H., Atkins, D., Ritter, D., Simon, R., Laffel, L., & Lipman, T. H. (2022). A text messaging intervention with financial incentive for adolescents with type 1 diabetes. *Journal of Diabetes Science and Technology, 16*(1), 120–127. https://doi.org/10.1177/1932296820952786

14. Spaic, T., Robinson, T., Goldbloom, E., Gallego, P., Hramiak, I., Lawson, M. L., Malcolm, J., Mahon, J., Morrison, D., Parikh, A., Simone, A., Stein, R., Uvarov, A., Clarson, C., & JDRF Canadian Clinical Trial CCTN1102 Study Group (2019). Closing the gap: Results of the multicenter canadian randomized controlled trial of structured transition in young adults with type 1 diabetes. *Diabetes care*, *42*(6), 1018–1026. https://doi.org/10.2337/dc18-2187

15. Petrovski, G., & Zivkovic, M. (2017). Impact of facebook on glucose control in type 1 diabetes: A three-year cohort study. *JMIR Diabetes*, *2*(1), e9. https://doi.org/10.2196/diabetes.7693

16-1. Wagner, J. A., Petry, N. M., Weyman, K., Tichy, E., Cengiz, E., Zajac, K., & Tamborlane, W. V. (2019). Glucose management for rewards: A randomized trial to improve glucose monitoring and associated self-management behaviors in adolescents with type 1 diabetes. *Pediatric Diabetes*, *20*(7), 997–1006. https://doi.org/10.1111/pedi.12889

16-2. Wong, J. J., Addala, A., Naranjo, D., Hood, K. K., Cengiz, E., Ginley, M. K., ... & Wagner, J. A. (2020). Monetary reinforcement for self‐monitoring of blood glucose among young people with type 1 diabetes: Evaluating effects on psychosocial functioning. *Diabetic Medicine*, *37*(4), 665-673. http://doi.org/10.1111/dme.14174

17. Jaser, S. S., Whittemore, R., Choi, L., Nwosu, S., & Russell, W. E. (2019). Randomized trial of a positive psychology intervention for adolescents with type 1 diabetes. *Journal of Pediatric Psychology*, *44*(5), 620–629. https://doi.org/10.1093/jpepsy/jsz006

18. Al Ksir, K., Wood, D. L., Hasni, Y., Sahli, J., Quinn, M., & Ghardallou, M. (2022). Motivational interviewing to improve self-management in youth with type 1 diabetes: A randomized clinical trial. *Journal of Pediatric Nursing*, *66*, e116–e121. https://doi.org/10.1016/j.pedn.2022.05.001

19. Brorsson, A. L., Leksell, J., Andersson Franko, M., & Lindholm Olinder, A. (2019). A person-centered education for adolescents with type 1 diabetes-a randomized controlled trial. *Pediatric Diabetes*, *20*(7), 986–996. https://doi.org/10.1111/pedi.12888

20. Tuomaala, A. K., Hero, M., Tuomisto, M. T., Lähteenmäki, M., Miettinen, P. J., Laine, T., Wehkalampi, K., Kiiveri, S., Ahonen, P., Ojaniemi, M., Kaunisto, K., Tossavainen, P., Lapatto, R., Sarkola, T., & Pulkkinen, M. A. (2021). Motivational interviewing and glycemic control in adolescents with poorly controlled type 1 diabetes: A randomized controlled pilot trial. *Frontiers in Endocrinology*, *12*, 639507. https://doi.org/10.3389/fendo.2021.639507

21. Cook, S., Herold, K., Edidin, D. V., & Briars, R. (2002). Increasing problem solving in adolescents with type 1 diabetes: The choices diabetes program. *Diabetes Education & Training,* *28*(1), 115–124. https://doi.org/10.1177/014572170202800113

22. Fiallo-Scharer, R., Palta, M., Chewning, B. A., Rajamanickam, V., Wysocki, T., Wetterneck, T. B., & Cox, E. D. (2019). Impact of family-centered tailoring of pediatric diabetes self-management resources. *Pediatric Diabetes, 20*(7), 1016–1024. https://doi.org/10.1111/pedi.12899

23. Hannon, T. S., Yazel-Smith, L. G., Hatton, A. S., Stanton, J. L., Moser, E. A. S., Li, X., & Carroll, A. E. (2018). Advancing diabetes management in adolescents: Comparative effectiveness of mobile self-monitoring blood glucose technology and family-centered goal setting. *Pediatric Diabetes*, *19*(4), 776–781. https://doi.org/10.1111/pedi.12648

24. Mayer-Davis, E. J., Maahs, D. M., Seid, M., Crandell, J., Bishop, F. K., Driscoll, K. A., Hunter, C. M., Kichler, J. C., Standiford, D., Thomas, J. M., & FLEX Study Group (2018). Efficacy of the flexible lifestyles empowering change intervention on metabolic and psychosocial outcomes in adolescents with type 1 diabetes (flex): A randomized controlled trial. *The Lancet Child & Adolescent Health,* *2*(9), 635–646. https://doi.org/10.1016/S2352-4642(18)30208-6

25. Murphy, H. R., Wadham, C., Hassler-Hurst, J., Rayman, G., Skinner, T. C., & Families and Adolescents Communication and Teamwork Study (FACTS) Group (2012). Randomized trial of a diabetes self-management education and family teamwork intervention in adolescents with type 1 diabetes. *Diabetic Medicine*, *29*(8), e249–e254. https://doi.org/10.1111/j.1464-5491.2012.03683.x

26-1. Bisno, D. I., Reid, M. W., Pyatak, E. A., Flores Garcia, J., Salcedo-Rodriguez, E., Torres Sanchez, A., Fox, D. S., Hiyari, S., Fogel, J. L., Marshall, I., Bachmann, G., & Raymond, J. K. (2023). Virtual peer groups reduce HbA1c and increase continuous glucose monitor use in adolescents and young adults with type 1 diabetes. *Diabetes Technology & Therapeutics,* *25*(9), 589–601. https://doi.org/10.1089/dia.2023.0199

26-2. Garcia, J. F., Faye, E., Reid, M. W., Pyatak, E. A., Fox, D. S., Bisno, D. I., Salcedo-Rodriguez, E., Sanchez, A. T., Hiyari, S., Fogel, J. L., & Raymond, J. K. (2023). Greater telehealth use results in increased visit frequency and lower physician related-distress in adolescents and young adults with type 1 diabetes. *Journal of Diabetes Science and Technology, 17*(4), 878–886. https://doi.org/10.1177/19322968221146806

27-1. Hood, K. K., Iturralde, E., Rausch, J., & Weissberg-Benchell, J. (2018). Preventing diabetes distress in adolescents with type 1 diabetes: Results 1 year after participation in the steps program. *Diabetes Care, 41*(8), 1623–1630. https://doi.org/10.2337/dc17-2556

27-2. Shapiro, J. B., Bryant, F. B., Holmbeck, G. N., Hood, K. K., & Weissberg-Benchell, J. (2021). Do baseline resilience profiles moderate the effects of a resilience-enhancing intervention for adolescents with type I diabetes? *Health Psychology*, *40*(5), 337–346. https://doi.org/10.1037/hea0001076

27-3. Iturralde, E., Weissberg-Benchell, J., & Hood, K. K. (2017). Avoidant coping and diabetes-related distress: Pathways to adolescents' type 1 diabetes outcomes. *Health Psychology*, *36*(3), 236–244. https://doi.org/10.1037/hea0000445

28. Jaser, S. S., Patel, N., Rothman, R. L., Choi, L., & Whittemore, R. (2014). Check it! a randomized pilot of a positive psychology intervention to improve adherence in adolescents with type 1 diabetes. *The Diabetes Educator*, *40*(5), 659–667. https://doi.org/10.1177/0145721714535990

29. McGill, D. E., Laffel, L. M., Volkening, L. K., Butler, D. A., Levy, W. L., Wasserman, R. M., & Anderson, B. J. (2020). Text message intervention for teens with type 1 diabetes preserves HbA1c: Results of a randomized controlled trial. *Diabetes Technology & Therapeutics, 22*(5), 374–382. https://doi.org/10.1089/dia.2019.0350

30. Channon, S. J., Huws-Thomas, M. V., Rollnick, S., Hood, K., Cannings-John, R. L., Rogers, C., & Gregory, J. W. (2007). A multicenter randomized controlled trial of motivational interviewing in teenagers with diabetes. *Diabetes Care*, *30*(6), 1390–1395. https://doi.org/10.2337/dc06-2260

31. Husted, G. R., Thorsteinsson, B., Esbensen, B. A., Gluud, C., Winkel, P., Hommel, E., & Zoffmann, V. (2014). Effect of guided self-determination youth intervention integrated into outpatient visits versus treatment as usual on glycemic control and life skills: A randomized clinical trial in adolescents with type 1 diabetes. *Trials*, *15*, 321. https://doi.org/10.1186/1745-6215-15-321

32. Boardway, R. H., Delamater, A. M., Tomakowsky, J., & Gutai, J. P. (1993). Stress management training for adolescents with diabetes. *Journal of Pediatric Psychology*, *18*(1), 29–45. https://doi.org/10.1093/jpepsy/18.1.29

33. Pulkkinen, M. A., Tuomaala, A. K., Hero, M., Gordin, D., & Sarkola, T. (2020). Motivational interview to improve vascular health in adolescents with poorly controlled type 1 Diabetes: A randomized controlled trial. *BMJ Open Diabetes Research & Care,* *8*(1), e001216. https://doi.org/10.1136/bmjdrc-2020-001216

34. Ellis, D. A., Frey, M. A., Naar-King, S., Templin, T., Cunningham, P., & Cakan, N. (2005). Use of multisystemic therapy to improve regimen adherence among adolescents with type 1 diabetes in chronic poor metabolic control: A randomized controlled trial. *Diabetes Care*, *28*(7), 1604–1610. https://doi.org/10.2337/diacare.28.7.1604

35. Ellis, D. A., Templin, T., Naar-King, S., Frey, M. A., Cunningham, P. B., Podolski, C. L., & Cakan, N. (2007). Multisystemic therapy for adolescents with poorly controlled type I diabetes: Stability of treatment effects in a randomized controlled trial. *Journal of Consulting and Clinical Psychology, 75*(1), 168–174. https://doi.org/10.1037/0022-006X.75.1.168

36. Whittemore, R., Grey, M., Lindemann, E., Ambrosino, J., & Jaser, S. (2010). Development of an internet coping skills training program for teenagers with type 1 diabetes. *Computers, Informatics, Nursing: CIN,* *28*(2), 103–111. https://doi.org/10.1097/NCN.0b013e3181cd8199

37-1. Stanger, C., Lansing, A. H., Scherer, E., Budney, A., Christiano, A. S., & Casella, S. J. (2018). A web-delivered multicomponent intervention for adolescents with poorly controlled type 1 diabetes: A pilot randomized controlled trial. *Annals of Behavioral Medicine*, *52*(12), 1010–1022. https://doi.org/10.1093/abm/kay005

37-2. Lansing, A. H., Stoianova, M., & Stanger, C. (2019). Adolescent emotional control moderate benefits of a multicomponent intervention to improve type 1 diabetes adherence: A pilot randomized controlled trial. *Journal of Pediatric Psychology,* *44*(1), 126–136. https://doi.org/10.1093/jpepsy/jsy071

38. de Wit, M., Delemarre-van de Waal, H. A., Bokma, J. A., Haasnoot, K., Houdijk, M. C., Gemke, R. J., & Snoek, F. J. (2008). Monitoring and discussing health-related quality of life in adolescents with type 1 diabetes improve psychosocial well-being: A randomized controlled trial. *Diabetes Care*, *31*(8), 1521–1526. https://doi.org/10.2337/dc08-0394

39. Wang, Y. C., Stewart, S. M., Mackenzie, M., Nakonezny, P. A., Edwards, D., & White, P. C. (2010). A randomized controlled trial comparing motivational interviewing in education to structured diabetes education in teens with type 1 diabetes. *Diabetes Care*, *33*(8), 1741–1743. https://doi.org/10.2337/dc10-0019

40. Olmsted, M. P., Daneman, D., Rydall, A. C., Lawson, M. L., & Rodin, G. (2002). The effects of psychoeducation on disturbed eating attitudes and behavior in young women with type 1 diabetes mellitus. *International Journal of Eating Disorders*, *32*(2), 230–239. <https://doi.org/10.1002/eat.10068>

**Appendix S4**

*Assessment of Risk of Bias in Included Studies*


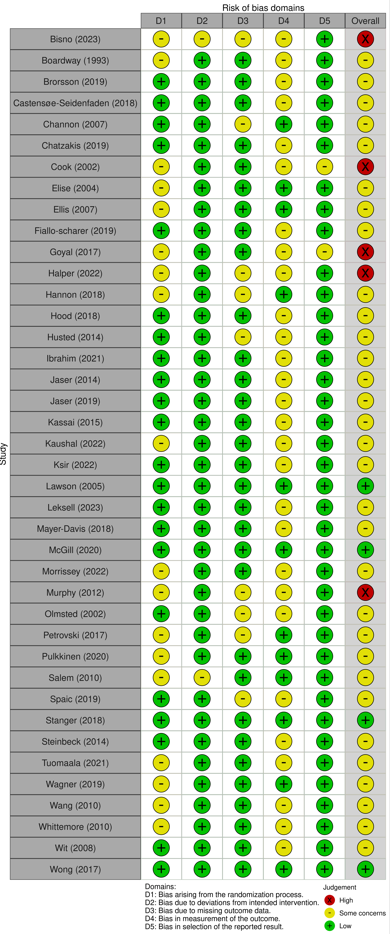


**Appendix S5**

*Funnel Plots of Publication Bias for HbA1c*


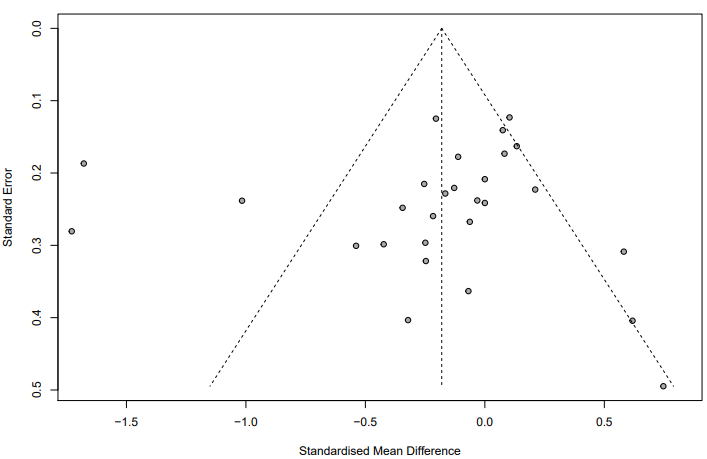


**Appendix S6**

*Sensitivity Analysis Results for Non-Pharmacological Intervention on Hba1c In 6-Month Follow-Up*

**Appendix S6-1** Forest plot of the effect of non-pharmacological interventions on HbA1c in 6-month follow-up after sensitivity analysis

| 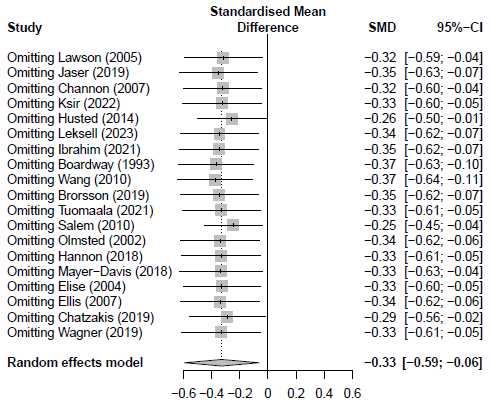  **1** |
| --- |

**Appendix S6-2** Baujat plot for sensitivity analysis of non-pharmacological interventions on HbA1c in 6-month follow-up

| 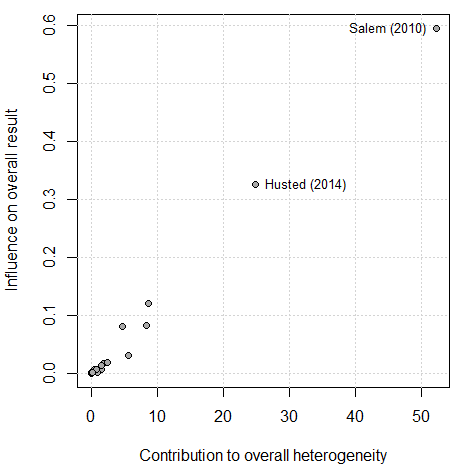  **2** |
| --- |

**Appendix S7**

*Sensitivity Analysis Results for Non-Pharmacological Intervention on HbA1c in 7-12 Month Follow-Up*

**Appendix S7-1** Forest plot of the effect of non-pharmacological interventions on HbA1c in 7-12month follow-up after sensitivity analysis

| 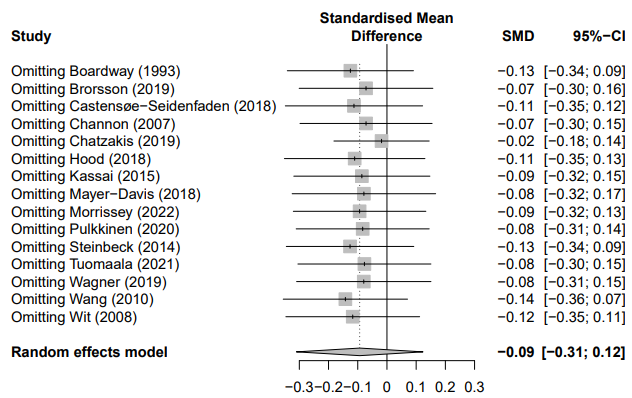  **1** |
| --- |

**Appendix S7-2** Baujat plot for sensitivity analysis of non-pharmacological interventions on HbA1c in follow-up 7~12months

| 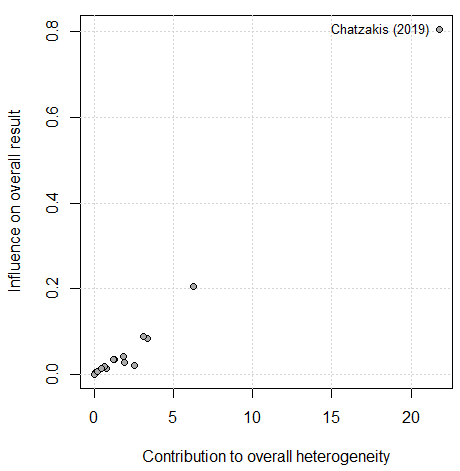  **2** |
| --- |

**Appendix S8**

*Sensitivity Analysis Results for Non-Pharmacological Intervention on HbA1c in* *13-Month Follow-Up*

**Appendix S8-1**. Forest plot of the effect of non-pharmacological interventions on HbA1c in 13-month follow-up after sensitivity analysis

| 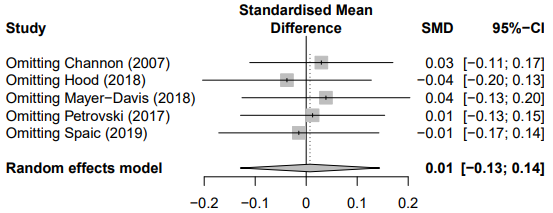  **1** |
| --- |

**Appendix S8-2**. Baujat plot for sensitivity analysis of non-pharmacological interventions on HbA1c in 13-month follow-up

| 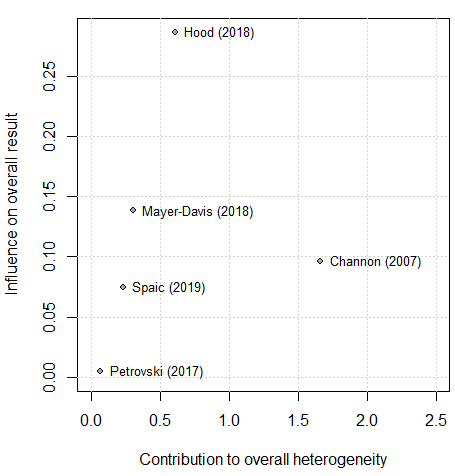  **2** |
| --- |
