# Supplementary material for: Long‐term effects of non‐pharmacological interventions in adolescents and young adults with type 1 diabetes: A systematic review and meta‐analysis
Source: Worldviews Evid Based Nurs. 2024 Nov 4;21(6):665–77. doi: 10.1111/wvn.12751 (PMC11655730; doi:10.1111/wvn.12751)
Supplement: Supplementary file 2 — Table S1 [file WVN-21-665-s002.docx]

**Table S1**

*Descriptive Summary of Included Studies*

| **No.** | **Author (year), country** | **Sample size**  **(mean age)** | | **Duration of T1D (years)** | **Ethnicity/race (sample size)** | **Intervention** | **Program duration (frequency)** | **Intervention duration (follow-up)** | **Control group** | **Outcome variables** |
| --- | --- | --- | --- | --- | --- | --- | --- | --- | --- | --- |
| **self-care behavior management** | | | | | | | | | | |
| 1 | Leksell (2023), Sweden | I: 35 (19.80)  C: 44 (20.70) | | NR | NR | ⦁Facilitates seamless real-time communication for participants  ⦁Text message exchanges  ⦁Schedule online appointments  ⦁Initiate impromptu video meetings | Access on demand | 6-month  (6-month) | No intervention | ⦁HbA1c  ⦁Time in range  ⦁Time below range  ⦁Satisfaction  ⦁QoL^a^ |
| 2 | Ibrahim (2021), France | I: 45 (15.39)  C: 47 (14.59) | | I: 8.70  C: 7.79 | NR | ⦁Received daily SMS reminders at self-selected times about insulin injections | 1-4 message per day | 6-month (3, 6-moth) | Standard care | ⦁HbA1c ^a^  ⦁QoL  ⦁Satisfaction  ⦁Adverse events |
| 3 | Halper (2022), USA | I (A): 34 (14.90)  I (B): 30 (15.10)  C: 35 (14.70) | | NR | African American: 9  White: 85  Other: 5 | ⦁A: Family-centered goal setting  ⦁B: health information technology (HIT)-enhanced self-monitoring of blood glucose (SMBG) strategy+family-centered goal setting | Every 3-months (goal setting), daily (SMBG) | 6-month (3, 6-month) | HIT-enhanced SMBG | ⦁Burden  ⦁QoL  ⦁Sleep quality  ⦁Adherence ^a^  ⦁Satisfaction ^a^ |
| 4 | Kassai (2015), France | I: 39 (14.30)  C: 38 (14.60) | | NR | NR | ⦁Nurse counseling  ⦁Pediatrician visit  ⦁Nurse visit intercalated every month  ⦁Phone calls | Every 2 weeks after each visit (phone), every  3 months (clinic) | 12-month (3, 6, 9, 12) | Pediatrician visit only every  3 months | ⦁HbA1c  ⦁Acceptance of the disease |
| 5 | Salem (2010), Egypt | I (A): 75 (14.7)  I (B): 73 (14.5)  C: 48 (15.00) | | I (A):3.60  I (B): 5.50  C: 4.90 | NR | ⦁A: attended the exercise sessions once/week  ⦁B: attended the exercise sessions three times/week | once/week, three times/week | 6-month (6-month) | No intervention | ⦁HbA1c ^a^  ⦁Weight ^a^  ⦁BMI ^a^  ⦁Waist circumference ^a^  ⦁Bp ^a^  ⦁Insulin dosage  ⦁Adverse events  ⦁Lipid profile ^a^ |
| 6 | Morrissey (2022), Ireland | I (A): 23 (20.30)  I (B): 25 (20.70)  C: 9 (20.60) | | NR | NR | ⦁Support self-management and clinic engagement and improve outcomes  ⦁A: D1 now intervention with the external support worker  ⦁B: D1 now intervention with the internal support-worker  ⦁Focuses on promoting engagement of young people with clinical services  ⦁Provide continuity and build relationships between the young adult and their healthcare team | Every 4-months | 12-month (12-month) | Usual care | ⦁HbA1c  ⦁Adverse events  ⦁Clinic engagement  ⦁Distress  ⦁QoL  ⦁Self-care  ⦁Control of diabetes |
| 7 | Chatzakis (2019), Greece | I: 40. (13.80)  C: 40 (13.20) | | I: 6.70  C: 6.10 | NR | ⦁Mobile Application Euglyca (calculation of the bolus insulin dose) | Daily | 12-month (3, 6, 12-month) | Routine calculation | ⦁HbA1c ^a^  ⦁Diabetes treatment satisfaction ^a^  ⦁Percentages of normoglycemias ^a^  ⦁Adverse events |
| 8 | Goyal (2017), Canada | I: 46 (14.10)  C: 46 (13.90) | | I: 7.10  C: 7.04 | NR | ⦁Users were rewarded for taking SMBG  ⦁Maintaining their blood glucose within their target range | Daily text message | 12-month (3, 6, 9, 12-month) | Usual clinical care | ⦁HbA1c  ⦁Frequency of BGM  ⦁Adverse events ^a^  ⦁QoL  ⦁Family responsibility  ⦁Self-care  ⦁Readiness |
| 9 | Wong (2017), USA | I: 45 (16.00)  C:45 (16.50) | | NR | African American: 10  Hispanic: 11  White: 64  Other non-Hispanic: 5 | ⦁Daily financial incentives  ⦁Daily monitoring, incentive feedback | Four or more blood glucose checks per day | 3-month (3-month) | No intervention | ⦁HbA1c  ⦁Frequency of BGM |
|  | | | **Combined with self-care behavior management and education** | | | | | | | |
| 10 | Castensøe-Seidenfaden (2018), Denmark | I: 76 (17.60)  C: 75 (17.60) | | I: 8.30  C: 7.70 | NR | **mHealth app:** 8 main functions (My Page, My Department, Chat Room, Carbohydrate Counting, Information about..., Tips Package, To Parents, Reminder Function) | Download in 10 min, 1 hour (introduction app) | 12-month (2, 7, 12-month) | Usual care | ⦁HbA1c ^a^  ⦁Competence  ⦁Health care climate  ⦁Distress  ⦁Severe hypoglycemic episodes  ⦁Acute diabetes-related hospitalizations |
| 11 | Lawson (2005), Canada | I: 23 (15.40)  C: 23 (15.00) | | I: 6.70  C: 6.30 | NR | ⦁Phone call (adolescents’ lives and diabetes education, blood glucose results and insulin-dose adjustment) | Once/wk | 6-month (3, 6-month) | Standard care | ⦁HbA1c  ⦁Compliance with glucose monitoring  ⦁QoL  ⦁Family functioning  ⦁Daily insulin dose  ⦁BMI ^a^  ⦁Adverse events |
| 12 | Steinbeck (2014), Australia | I: 14 (18.08)  C: 12 (17.70) | | I: 8.36  C: 9.06 | NR | **Post-discharge program**  ⦁Telephone communication support | 1 wk, 3, 6, 12-month, 2-20 min (phone call) | 12-month | Standard clinical  practice | ⦁HbA1c ^a^  ⦁Diabetes-related hospitalizations  ⦁Microvascular complication appearance  ⦁Self-worth |
| 13 | Kaushal (2022), USA | I: 83 (15.80)  C: 82 (15.50) | | I: 7.20  C: 7.00 | African American: 46  Asian: 2  Multiracial: 11  White: 97  Other: 9 | ⦁Incentivized text messaging intervention  ⦁Received a combination of declarative text messages about their pre-selected self-care behavior goal | Daily | 6-month (3, 6-month) | Standard care | ⦁HbA1c  ⦁Self-care |
| 14 | Spaic (2019), Canada | I: 104 (17.90)  C: 101 (17.90) | | I: 9.40  C: 10.10 | Asian or pacific islander: 6  White: 175  African American: 11  Aboriginal: 0  Other: 13 | ⦁Transition program  ⦁Transition coordinator  ⦁Specific transition-related education and education materials | Six-visit during 18-month | 18-month (12-month) | Standard care | ⦁HbA1c  ⦁Satisfaction  ⦁Distress  ⦁QoL |
| 15 | Petrovski (2017), Macedonia | I: 27 (17.40)  C: 29 (16.90) | | I: 5.40  C: 5.60 | NR | ⦁Social Media  ⦁CareLink personal program  ⦁Intervention (education, pump settings, basal and bolus insulin) | NR | 36-month (3, 6, 9, 12, 15, 18-moth) | Regular group (treated using standard medical protocol with regular visits at clinic) | ⦁HbA1c ^a^  ⦁Blood glucose ^a^  ⦁Adverse events |
| 16(1) | Wagner (2019), USA ^a^ | I: 32 (15.30±2.20)  C: 28 (15.90±2.50)  Inclusion criteria of age: 12-21 | | I: 7.00±4.10  C: 7.40±4.70 | African American: 6  White: 48  Other: 6 | ⦁Enhanced usual care+earning the following monetary reinforcements  ⦁SMBG education, SMBG plan, upload SMBG data  ⦁Conducting SMBG four times daily, texting SMBG updates, uploading glucose meters, and responding promptly to clinician questions about glucose patterns | 30-min (education), upload weekly or more often (SMBG) | 26-week (9-month) | Enhanced usual care | ⦁HbA1c ^a^  ⦁Frequency of BGM ^a^ |
| 16(2) | Wong (2019), USA |  | |  |  |  |  |  |  | ⦁Distress ^a^  ⦁Family conflict  ⦁QoL ^a^  ⦁Affective response |
| **Combined with self-care behavior management, education and psychological intervention** | | | | | | | | | | |
| 17 | Jaser (2019), USA | I(A+B): 60 (14.78)  C: 60 (14.88) | | I(A+B): 5.47  C: 6.22 | White: 105  Other: 14  Unknown: 1 | **Positive psychology (PA) intervention**  ⦁T1dm+caregiver  ⦁Positive Affect (PA) interview  ⦁Educational material  ⦁Health behavior contract, in which they identified a goal for increasing frequency of BGM  ⦁I (A): text message  ⦁I (B): phone call | Educational material twice/wk (8 wk), Text message once/wk (8 wk), phone call weekly once/wk (8 wk) | 8-week (3, 6-month) | Education | ⦁HbA1c  ⦁Frequency of BGM  ⦁QoL  ⦁Self-care  ⦁Positive and negative affect  ⦁Stress |
| 18 | Ksir (2022), Tunisia | I: 33 (15.30)  C: 33 (15.06) | | I: 6.64  C: 4.30 | NR | ⦁Motivational interviewing (MI)  ⦁Nurse-led education program based on motivational interviewing  ⦁To support diabetes specific self-management skills  ⦁Educational materials | 2-session, 20-30 min per session, 4-phone call, 10 min per call every month | 6-month (3, 6-month) | Usual care | ⦁HbA1c ^a^  ⦁Transition readiness ^a^ |
| 19 | Brorsson (2019), Sweden | I: 37 (14.80)  C: 32 (15.10) | | I: 4.50  C: 5.60 | NR | ⦁Group-based standard insulin pump introduction program  ⦁Educational group sessions  ⦁Specific reflection worksheets | 7-group session, 2 hr per session, program held 4-5 day | 5-month (6, 12-month) | Standard care | ⦁HbA1c ^a^  ⦁Family conflicts  ⦁Burden  ⦁QoL  ⦁Self-efficacy |
| 20 | Tuomaala (2021), Finland | I: 24 (14.60)  C: 23 (14.60) | | I: 8.30  C: 7.80 | NR | ⦁MI+standard education (SE)  ⦁Improving adherence to glucose monitoring and insulin administration | Every 3-months | 12-month (3, 6, 9, 12-month) | SE | ⦁HbA1c ^a^  ⦁Time in range  ⦁Glycemic variability  ⦁QoL |
| 21 | Cook (2002), USA | I: 26 (14.80)  C: 27 (14.40) | | NR | African American: 2  Hispanic: 4  White: 45 | ⦁Problem-solving diabetes education program  ⦁Education booklets  ⦁To discuss major problems  ⦁Specific behavioral and cognitive skills | Once a week | 6-week (6-month) | Usual care | ⦁HbA1c  ⦁Diabetes problem-solving  ⦁Diabetes behavior  ⦁Frequency of BGM ^a^ |
| 22 | Fiallo-Scharer (2019), USA | Site 1, age 13-16  I: 25  C: 23  Site 2, age 13-16  I: 36  C: 35 | | Site 1  I: 7.00  C: 6.60  Site 2  I: 5.70  C: 5.70 | Site 1  White: 44  All other: 4  Site 2  White: 56  All other: 15 | ⦁Family-centered approach  ⦁Identified barriers  ⦁Tailored self-management methods  ⦁Group session content and all materials  ⦁MI | 4-session, 75 min per session | 12-month (15, 18, 21, 24-month) | Usual care | ⦁HbA1c ^a^  ⦁QoL |
| 23 | Hannon (2018), Indiana | I (A): 33  I (B): 31  C: 33  Age mean included in study: 14.80±1.60 | | I (A): 5.00  I (B): 6.10  C: 5.80 | African American: 8  Asian: 1  Hispanic or Latino: 5  Multiracial: 3  White: 80 | ⦁I (A): Family-centered goal setting  ⦁I (B): health information technology (HIT) enhanced SMBG strategy + family-centered goal setting  ⦁Setting family-centered goals using MI (frequency of SMBG, bolus insulin, self-adjustment of insulin, frequency of contact with the diabetes care team) | Weekly (reviewed all SMBG data), visit every 3-months (30-min) | 6-month (3, 6-month) | HIT-enhanced SMBG | ⦁HbA1c  ⦁Frequency of BGM |
| 24 | Mayer-Davis (2018), USA | I: 130 (14.80)  C: 128 (14.90) | | I: 6.48  C: 6.39 | African American: 11  Hispanic: 33  White: 200  Other: 14 | ⦁MI  ⦁FLEX-basic (introduced concepts of family communication and teamwork)  ⦁FLEX toolbox (diabetes education, social support, use of communication technology)  ⦁FLEX regular or FLEX check-in (session after FLEX-basic) | 4-session (12 wk), 40-60 min per session, coach met 3-4 time (every 6-month), 10-15 min phone call per month | 18-month (3, 6, 12, 18-moth) | Usual care | ⦁HbA1c  ⦁Motivation  ⦁Intention  ⦁Problem solving ^a^  ⦁Self-care ^a^  ⦁Depression  ⦁QoL ^a^  ⦁Adverse events  ⦁Family conflict  ⦁BMI  ⦁Blood lipids ^a^  ⦁Bp ^a^ |
| 25 | Murphy (2012), UK | I: 158  C: 147  Mean age of all: 13.1±1.90 | | Mean diabetes duration of all: 5.60 | NR | ⦁Conventional diabetes self-management education  ⦁Incorporating skills training  ⦁Family communication training  ⦁Small groups (4–6 families per group) | 6-session, 90-min (monthly) | 6-month (3, 6, 9, 12-month) | Conventional clinical care | ⦁HbA1c  ⦁QoL  ⦁Well-being  ⦁Distress (parent)  ⦁Family responsibility  ⦁Proactivity in adjusting insulin ^a^  ⦁Adverse events |
| 26(1) | Bisno (2023), USA | I (A): 19 (18.44)  I (B): 21 (18.29)  C: 28 (17.82) | | I (A): 8.89  I (B): 9.29  C: 6.84 | Aboriginal: 8  Asian: 4  African American: 6  Multiracial: 8  White: 29  Unknown: 13 | **CoYoT1 care**  ⦁Person-centered, home tele-health care model  ⦁Virtual peer group (VPG): discussion group, encouraging self-disclosure, problem solving and emotional support  ⦁Video conference  ⦁Adolescent and young adult-focused (AYA): diabetes distress and burnout, managing diabetes at college, social and intimate relationships with diabetes, diabetes research and technology, drinking and diabetes, and how to manage diabetes when life gets busy | 2-meeting (family focused), 6-meeting (AYA-focused), visits every 3 months | 15-month (3, 6, 12-month) | Standard care | ⦁HbA1c ^a^  ⦁Diabetes empowerment  ⦁Depression  ⦁Distress ^a^ |
| 26(2) | Garcia (2023), USA | I: 39 (17.97)  C: 29 (18.38) | | I: 8.16  C: 8.13 |  | >50.0% visits via telehealth |  |  | >50.0% visits via in-persion | ⦁HbA1c  ⦁Self-efficacy  ⦁Distress ^a^ |
| 27(1) | Hood (2018) USA | I: 133  C: 131  Mean age of all: 15.74 | | Mean diabetes duration of all: 6.88 | Aboriginal: 3  Asian or pacific islander: 6  African American: 38  Hispanic: 29  White: 173  Other: 15 | **Supporting Teen Problem-Solving**  ⦁Penn resilience program curriculum (T1D specific challenges and examples)  ⦁Intervention-specific student workbook  ⦁Group discussion and interaction throughout | 9-session, 90-120 min per session (every other week | 4.5-month (4.5, 8, 12, 16-moth) | Education intervention | ⦁HbA1c  ⦁Self-care  ⦁Resilience  ⦁Distress ^a^  ⦁Depression |
| 27(2) | Shapiro (2022), USA |  | |  |  |  |  |  |  | ⦁HbA1c  ⦁Self-efficacy  ⦁Social problem solving  ⦁Negative automatic thoughts  ⦁Hopelessness  ⦁Family conflict  ⦁Distress  ⦁Self-care  ⦁Frequency of BGM |
| 27(3) | Iturralde (2017), USA |  | |  |  |  |  |  |  | ⦁HbA1c  ⦁Distress  ⦁Frequency of BGM  ⦁Avoidant coping style |
| 28 | Jaser (2014), USA | I: 20 (15.30)  C: 19 (15.00) | | I: 7.30  C: 6.50 | White: 30  Non-white: 8 | **Positive psychology intervention**  ⦁ Positive affect interview  ⦁Phone calls (reminded to use gratitude and self-affirmation)  ⦁Affirmations to adolescents on topics about diabetes self-care (parents) | Phone call twice/wk (8 wk), weekly affirmations (parent) | 8-week (3, 6-month) | Education | ⦁HbA1c  ⦁Frequency of BGM  ⦁Self-care  ⦁Positive and negative affect  ⦁Depression  ⦁Family conflict  ⦁QoL |
|  | | | **Combined with self-care behavior management and psychological intervention** | | | | | | | |
| 29 | McGill (2020), USA | I (A): 74 (15.00)  I (B): 74 (14.90)  I (A+B): 77 (14.09)  C: 76 (15.10) | | I (A): 6.20  I (B): 7.30  I (A+B): 6.90  C: 5.80 | White: 234  Unknown: 67 | ⦁I (A): Teenwork (TW)  ⦁I (B): Text message  ⦁I (A+B): TW+text message  ⦁TW: Improving self-care using MI  ⦁Text: Text reminders to check blood glucose | Maximum of four texts daily | 12-month (3, 6, 9, 12-month) | Usual care | ⦁HbA1c  ⦁Frequency of BGM ^a^ |
| 30 | Channon (2007), UK | I: 38 (15.30)  C: 28 (15.40) | | I: 9.20  C: 9.10 | White (Caucasian): 66 | ⦁MI  ⦁Awareness building  ⦁Alternatives  ⦁Problem solving  ⦁Making choices  ⦁Goal setting  ⦁Avoidance of confrontation | 20-60 min | 12-month (6, 12, 24-month) | Non-directive psychological support | ⦁HbA1c  ⦁QoL ^a^  ⦁Child health locus of control  ⦁Health care climate  ⦁Knowledge  ⦁Self-efficacy  ⦁Well-being ^a^  ⦁Diabetes family behavior  ⦁Personal models of diabetes ^a^ |
| 31 | Husted (2014), Denmark | I: 37 (14.90)  C: 34 (14.60) | | I: 6.10  C: 5.30 | Danish: 56  Other: 15 | ⦁18 semi-structured reflection sheets  ⦁To express their individual and shared difficulties with diabetes when coming to outpatient sessions  ⦁Life skills training process | 8-session (1-hr) | 8-12-month (6-month) | Usual care | ⦁HbA1c  ⦁Competence  ⦁Health care climate  ⦁Self-regulation ^a^  ⦁Burden  ⦁Well-being  ⦁Perception (parent) |
| 32 | Boardway (1993), USA | I: 9 (15.44)  C: 10 (14.32) | | I: 6.92  C: 6.34 | African American: 4  Asian: 1  Hispanic: 1  White: 13 | ⦁Stress management training  ⦁Group behavioral intervention program  ⦁Dietary and insulin administration skills | 13-session during 6-month | 9-month (3, 6, 9-moth) | Standard care | ⦁HbA1c  ⦁Stress ^a^  ⦁Coping responses  ⦁Self-efficacy  ⦁Life events  ⦁Fructosamine level  ⦁Frequency of BGM  ⦁Behavioral change |
| 33 | Pulkkinen (2020), Finland | I: 20 (14.60)  C: 21 (14.60) | | I: 8.10  C: 7.90 | NR | ⦁MI+SE  ⦁SE: carb counting, HbA1c targets, blood glucose targets, long-term diabetes complications, ketoacidosis and hypoglycemia | NR | 12-month (12-month) | SE | ⦁HbA1c  ⦁Blood glucose  ⦁Pulse wave velocity  ⦁Vascular ultrasound  ⦁BMI  ⦁Body composition  ⦁Bp ^a^ |
| 34 | Ellis (2004), USA | I: 13  C: 12  Mean age of all: 13.60±1.60 | | NR | African American: 15  Other ethnic or racial background: 2  White: 8 | ⦁Multisystemic therapy  ⦁Family interventions  ⦁Peer intervention  ⦁Community intervention  ⦁Health care system | 46-session | 6-month (6-moth) | Standard care | ⦁HbA1c ^a^  ⦁Self-care  ⦁Self-care (parent)  ⦁Frequency of BGM  ⦁Adherence (insulin, dietary, blood glucose testing) ^a^  ⦁Emergency room visits  ⦁Hospital admissions ^a^ |
| 35 | Ellis (2007), USA | I: 64 (13.40)  C: 63 (13.10) | | NR | African American: 80  Others (biracial and Hispanic): 14  White: 33 | ⦁Multisystemic Therapy  ⦁Focused on addressing adherence-related problems within both the family and broader community systems, targeting factors and communication gaps | Initial 5-day training, weekly on-site clinical supervision, weekly phone consultation with an MST expert, quarterly booster training, | 7-month (6-month) | Standard medical care | ⦁HbA1c  ⦁Frequency of BGM  ⦁Hospital admissions |
| 36 | Whittemore (2010), USA | I: 6  C: 6  Mean age of all: 14.40 | | Mean duration of all: 5.90 | Hispanic: 1  White: 11 | ⦁Web site  ⦁Session: self-talk, communication skills, social problem skills, stress management, and conflict  ⦁Managing diabetes: glucose control, nutrition, exercise and sick days, and new technology | 5-sessions (weekly) | 5-week (3, 6-month) | Managing Diabetes Internet interventions | ⦁HbA1c  ⦁QoL  ⦁Stress  ⦁Coping responses  ⦁Self-efficacy  ⦁Depressive  ⦁Satisfaction |
| 37(1) | Stanger (2018), USA ^a^ | I: 30 (15.20)  C: 31 (14.90) | | I: 5.90  C: 6.50 | Non-white or Hispanic: 2  White and non-Hispanic: 59 | ⦁Web-delivered multi-component intervention (self-monitoring of blood glucose, working memory, and parent supervision of diabetes care)  ⦁Incentives, MI, working memory training and contingency contracting sessions (parents) | 15-session (25 wk), 5 working memory training per week, once/wk (call) | 6-month (6, 12-month) | Usual care | ⦁HbA1c ^a^  ⦁Frequency of BGM ^a^  ⦁Visual spatial working memory ^a^  ⦁Family conflicts ^a^  ⦁Inhibitory control ^a^ |
| 37(2) | Lansing (2019), USA |  | |  |  |  |  |  |  | ⦁HbA1c  ⦁Frequency of BGM  ⦁Emotional control |
|  | | | **Combined with education and psychological intervention** | | | | | | | |
| 38 | Wit (2008), Netherland | I: 41 (14.80)  C: 40 (14.90) | | I: 7.20  C: 6.20 | Ethnic minority: 9  Unknown: 72 | **HRQoL intervention**  ⦁Monitoring  ⦁Discussion of the HRQoL scores with the teenager | 3-regular appointments at 3-month interval | 12-month (3, 6, 9-month) | Usual care | ⦁HbA1c  ⦁Well-being ^a^  ⦁Depression  ⦁Family conflicts  ⦁Satisfaction  ⦁QoL |
| 39 | Wang (2010), USA | I: 21 (15.30)  C: 23 (15.60) | | I: 6.70  C: 7.60 | White: 30  Other: 14 | MI–based education | 3-session | 3-month (3, 6, 9-moth) | Structured diabetes education | ⦁HbA1c ^a^  ⦁Depression  ⦁QoL  ⦁Self-care |
| 40 | Olmsted (2002), Canada | I: 50  C: 35  Age mean included in study (range): 16.00 (12-20) | | Mean duration of all: 7.00 | NR | ⦁Psychoeducation (PE) program  ⦁PE material  ⦁Written and oral presentation of didactic information | six-session per week, 90-min | 6-week (6-month) | Usual care | ⦁HbA1c  ⦁Eating disorder examination ^a^  ⦁Eating disorder inventory ^a^  ⦁Behavior related to eating and weight psychopathology  ⦁Insulin omission day |
| *Note*. AYA = adolescent and young adult-focused; BGM = blood glucose monitoring; BM = body max index; Bp = blood pressure; C = control group; FLEX = flexible lifestyles empowering change; HRQoL = health related quality of life; HIT = health information technology; I = intervention group; MI = motivational interview; NR = not reported; PA = positive affect; PE = psychoeducation; QoL = quality of life; SE = standard education; SMBG = self-monitoring of blood glucose; T1D = type 1 diabetes; VPG = virtual peer group.  ^a^ There was a statistically significant effect.  ^b^ (A) self-care behavior management, (B) education, (C) psychological intervention. | | | | | | | | | | |
